# Novel protein pathways in development and progression of pulmonary sarcoidosis

**DOI:** 10.1038/s41598-020-69281-8

**Published:** 2020-08-06

**Authors:** Maneesh Bhargava, K. J. Viken, B. Barkes, T. J. Griffin, M. Gillespie, P. D. Jagtap, R. Sajulga, E. J. Peterson, H. E. Dincer, L. Li, C. I. Restrepo, B. P. O’Connor, T. E. Fingerlin, D. M. Perlman, L. A. Maier

**Affiliations:** 1 Division of Pulmonary, Critical Care and Sleep Medicine, University of Minnesota, MMC 276, 420 Delaware St SE, Minneapolis, MN USA; 2 Division of Environmental and Occupational Health Sciences, National Jewish Health, Denver, CO USA; 3 Biochemistry, Molecular Biology and Biophysics, College of Biological Sciences, University of Minnesota, Minneapolis, MN USA; 4 Center for Immunology, University of Minnesota, Minneapolis, MN USA; 5 Center for Genes, Environment and Health, National Jewish Health, Denver, CO USA

**Keywords:** Proteome informatics, Pathogenesis, Biomarkers, Translational research, Molecular biology, Proteomics

## Abstract

Pulmonary involvement occurs in up to 95% of sarcoidosis cases. In this pilot study, we examine lung compartment-specific protein expression to identify pathways linked to development and progression of pulmonary sarcoidosis. We characterized bronchoalveolar lavage (BAL) cells and fluid (BALF) proteins in recently diagnosed sarcoidosis cases. We identified 4,306 proteins in BAL cells, of which 272 proteins were differentially expressed in sarcoidosis compared to controls. These proteins map to novel pathways such as integrin-linked kinase and IL-8 signaling and previously implicated pathways in sarcoidosis, including phagosome maturation, clathrin-mediated endocytic signaling and redox balance. In the BALF, the differentially expressed proteins map to several pathways identified in the BAL cells. The differentially expressed BALF proteins also map to aryl hydrocarbon signaling, communication between innate and adaptive immune response, integrin, PTEN and phospholipase C signaling, serotonin and tryptophan metabolism, autophagy, and B cell receptor signaling. Additional pathways that were different between progressive and non-progressive sarcoidosis in the BALF included CD28 signaling and PFKFB4 signaling. Our studies demonstrate the power of contemporary proteomics to reveal novel mechanisms operational in sarcoidosis. Application of our workflows in well-phenotyped large cohorts maybe beneficial to identify biomarkers for diagnosis and prognosis and therapeutically tenable molecular mechanisms.

## Introduction

Sarcoidosis is a multisystem immune-mediated disease of unknown cause with widely variable disease manifestations, severity, and outcomes^1^. It affects 45–300/100,000 individuals in the US, all ages, races, and both sexes^2,3^. Diagnostic delays are frequent as sarcoidosis is a diagnosis of exclusion, with no confirmatory test currently available. Despite a greater understanding of sarcoidosis pathogenesis^4,5^, the mechanisms contributing to the heterogeneity of disease manifestations and predictors of disease outcomes are poorly defined^6^. The annual mortality is approximately 2.8/million people^1^ and rising. Sarcoidosis-related mortality is attributed to four high-risk manifestations that include: treatment-resistant pulmonary sarcoidosis, multi-organ sarcoidosis, cardiac sarcoidosis, and neurosarcoidosis^7^. Respiratory failure from progressive pulmonary disease is the leading cause of sarcoidosis-related mortality in the US^7,8^. While remission is common, it is not known if systemic anti-inflammatory therapy decreases the risk of progressive pulmonary disease. Another current knowledge gap is the absence of validated markers to predict which patients with pulmonary sarcoidosis will progress.

The pathologic hallmark of sarcoidosis is the formation of epithelioid granuloma associated with infiltration of CD4 + T cells and scattered macrophages, giant cells, with CD8 + T cells and B cells around the granuloma^9^. While the exact details are not known, it appears that exposure to a yet unidentified antigen(s) results in an exuberant adaptive immune response with CD4 + T cells^10^, regulatory T cells (Tregs), and high levels of Th1 cytokines TNF–α, IFNγ, and IL–2. Additionally, an abnormal innate immune response is seen in bronchoalveolar lavage (BAL) cells in sarcoidosis. A less robust immune response is apparent in remitting disease compared to the exuberant response in progressive sarcoidosis, likely due to different T cell populations and abnormal counter-regulatory immune measures. Overall, the immune response is aberrant in sarcoidosis and compartmentalized to the lung with much higher response noted in the lung cells compared to blood cells^11,12^**.** The whole blood transcriptional profile of active sarcoidosis overlaps with tuberculosis and chronic beryllium disease, and inactive sarcoidosis overlaps with controls^13,14^. Genes with differential expression in sarcoidosis map to IFN-signaling, TLR signaling, and Fcɣ receptor-mediated phagocytosis^15,16^. In chronic progressive sarcoidosis, the gene expression in peripheral blood mononuclear cells demonstrates differential expression of genes participating in CXCL9 and TCR-mediated responses^17^. Transcriptional studies in BAL cells revealed that pathways linked to adaptive immune response, T-cell signaling, and chemokine signaling such as IFNγ, IL-12, 1L-17, and IL-23 are involved in sarcoidosis^18^. In lung tissue, gene networks engaged in cell movement, immune function, and in Th1-type responses such as signal transducer and activator of transcription 1 (STAT1), IL-5, IL-7, CXCR5, and CXCR9 were overexpressed in sarcoidosis lung tissues^11^. However, the approach of examining comprehensive protein changes that result from these differences in transcription is underutilized and has not been well evaluated using contemporary techniques.

Prior studies have used protein microarrays^19,20^, 2-dimensional electrophoresis (2DE)^12,21–23^, and top-down^24^ as well as shotgun proteomics^25–27^ to examine variable sarcoidosis phenotypes including Lofgren’s syndrome, non-Lofgren’s chest x-ray (CXR) stage I, and stage II/III pulmonary sarcoidosis and compared them to subjects with asthma, IPF, tuberculosis or healthy smoking and non-smoking controls. These studies have identified differences in protein spots on 2DE^12,21,22^, differentially expressed proteins^25,26,28^ and also possible mechanisms that could explain the development of sarcoidosis^25–27^. In a large study that utilized SELDI-TOF MS to compare BAL fluid (BALF) from sarcoidosis subjects with Lofgren’s syndrome and different CXR stages of pulmonary sarcoidosis (n = 65) with healthy controls, 40 differentially expressed peaks were identified compared to healthy controls and included 27 peaks that were specific for a particular CXR stage^24^. A study using affinity planar antigen microarray proteomics examining BALF and reported that mitochondrial ribosomal protein L43, nuclear receptor coactivator 2, adenosine diphosphate-ribosylation factor GTPase activating protein 1 and zinc finger protein 688 demonstrated higher reactivity in sarcoidosis lungs^20^. Another study reported several differentially expressed BALF proteins in nine sarcoidosis patients with stage II/III sarcoidosis compared to healthy controls analyzed by 2DE followed by MALDI-TOF MS^25^. The differentially expressed proteins mapped to canonical PI3K/Akt/mTOR signaling, MAP kinase, hypoxia response, and pluripotency-associated transactional factor pathways. These studies support rigorous evaluation of well-characterized, clinically-meaningful sarcoidosis phenotypes by contemporary techniques to identify novel mechanisms of sarcoidosis which can provide tenable treatment targets and biomarkers for personalized care.

Our goal is to couple contemporary proteomics with data-driven analytics for unbiased discovery of novel disease mechanisms in pulmonary sarcoidosis and progressive pulmonary disease, a known high-risk manifestation of sarcoidosis. As a critical first step in evaluating the proteome in sarcoidosis, we focus on BAL cells as alveolitis is seen in patients with active pulmonary sarcoidosis and immune cells provide an ex vivo model for biological mechanisms in inflammatory lung diseases. The BALF is the most proximate fluid to the site of injury, and thus has a high likelihood to identify disease-specific and potentially pathogenic changes. For this proof-of-concept study, we performed label-based MS for measuring protein abundance to gain insights into the intracellular protein interactions in sarcoidosis. We also employed label-free quantitative proteomics on BALF from controls and untreated sarcoidosis cases who, on follow-up, either were found to have progressive or non-progressive pulmonary disease. We found significant differences in BALF and cellular proteins between cases and controls and progressive versus non-progressive cases suggesting that this approach may find useful application in larger studies.

### Results

We characterized the proteins in BAL cells from four controls and four sarcoidosis cases. There was no difference in age, sex, race and smoking status for the two groups. The BAL leucocyte count was not significantly different but the sarcoidosis cases had more lymphocytes and a lower number of macrophages (Table 1). For the studies in BALF, we examined seven controls and ten sarcoidosis subjects (non-progressive = 5, progressive = 5) prior to initiation of any systemic anti-inflammatory therapy. There was no difference in the age, race, smoking status, BAL leucocytes, neutrophils, or lymphocytes and macrophages (Table 2). At enrollment, the forced vital capacity (FVC), forced expiratory volume in 1 second (FEV1) and diffusing capacity for carbon monoxide (DLCO) were also not different in subjects with progressive vs. non-progressive disease.

**Table 1:** Clinical and demographic variables for controls and sarcoidosis subjects for BAL cell studies.

	Controls (n = 4)	Sarcoidosis (n = 4)	p-value*
Age (years)	46 (39.5, 46.75)	39.5 (36, 46.75)	0.88
Sex (M/F)	2/2	2/2	1.00
Race (AA/C)	0/4	0/4	
Smoking (smokers/non-smokers)^#^	0/4	0/4	
BAL WBC count/μL	162 (122.5, 213.5)	83.5 (42.75, 166.3)	0.20
BAL neutrophils (%)	0.5 (0.27, 0.5)	1.0 (1.0, 1.75)	0.02
BAL lymphocytes (%)	5.1 (1.95, 9.375)	33.0 (15.0, 54.25)	0.03
BAL macrophages (%)	94.4 (90.35, 97.55)	65 (44.75, 83.0)	0.02
Percent predicted FVC		95 (84, 103.75)	
Percent predicted FEV1		93 (85.7, 99.2)	
Percent predicted DLCO		111 (94.5,121)	

Date presented as median (IQR).

*Mann-Whitney test or Chi-square test.

^#^All subjects were non-smokers (controls: 1 never smoker, 2 former smoker and 1 prior smoking history not known; cases: 3 never smokers and 1 former smoker).

**Table 2:** Clinical and demographic variables for controls and sarcoidosis subjects for BALF studies.

	Controls (n = 7)	Non-progressive (n = 5)	Progressive (n = 5)	p-value
Age (years)	32.0 (23.0, 54.0)	52.0 (41.0, 54.5)	53.0 (465, 54)	< 0.05*
Sex (M/F)	4/3	2/3	3/2	0.78
Race (AA/C)	0/7	1/4	0/5	0.28
Smoking (smokers/non-smokers)^#^	0/7	0/5	1/4	0.28
BAL WBC count/μL	105.5 (81.75, 163)	175 (120, 542)	80 (55, 112)	0.14
BAL neutrophils (%)	0.50 (0.2, 1.5)	0.50 (0.35, 1.3)	0.2 (0.1, 0.85)	0.56
BAL lymphocytes (%)	6.0 (4.2, 9.8)	6 (1.6, 40)	5 (3.5, 12)	0.21
BAL macrophages (%)	93 (90, 95)	94 (59, 98)	94 (87, 96)	0.22
Percent predicted FVC		91 (75, 100)	84 (71, 97)	0.55
Percent predicted FEV1		92.5 (70.25, 107.3)	68 (53, 86.5)	0.11
Percent predicted DLCO		93 (84.25, 97.25)	96 (93, 110.0)	0.40

Date presented as median (IQR).

ANOVA with post hoc Tukey test to compare all pairs of columns.

*Significant difference between controls and progressive group.

^#^All subjects except one were non-smokers (controls: 3 never smokers, 3 former smokers, 1 prior smoking history not known; non-progressive sarcoidosis: 3 never smokers, 2 former smokers; progressive sarcoidosis: 1 current smoker, 2 former smokers, 2 never smokers).

### Cellular proteins differ between sarcoidosis BAL cells and controls

The liquid chromatography (LC)-tandem mass spectrometry (MS/MS) identified 23,837 spectra at the given thresholds; 16,890 (71%) were included in quantitation. From these spectra, we identified 4,365 proteins (Supplemental Table S1; ‘Scaffold export’ tab). These included three proteins from the common Repository of Adventitious Proteins (cRAP) (serum albumin precursor, cluster of trypsin precursor and keratin, type 1 cytoskeletal 9) and 56 proteins that matched to the decoy (reverse) sequences, which were removed from further analysis resulting in identification of 4,306 high-confidence proteins (probability of 99%, Supplemental Table S2; ‘Scaffold-cleaned up’ tab).

**Figure 1:**
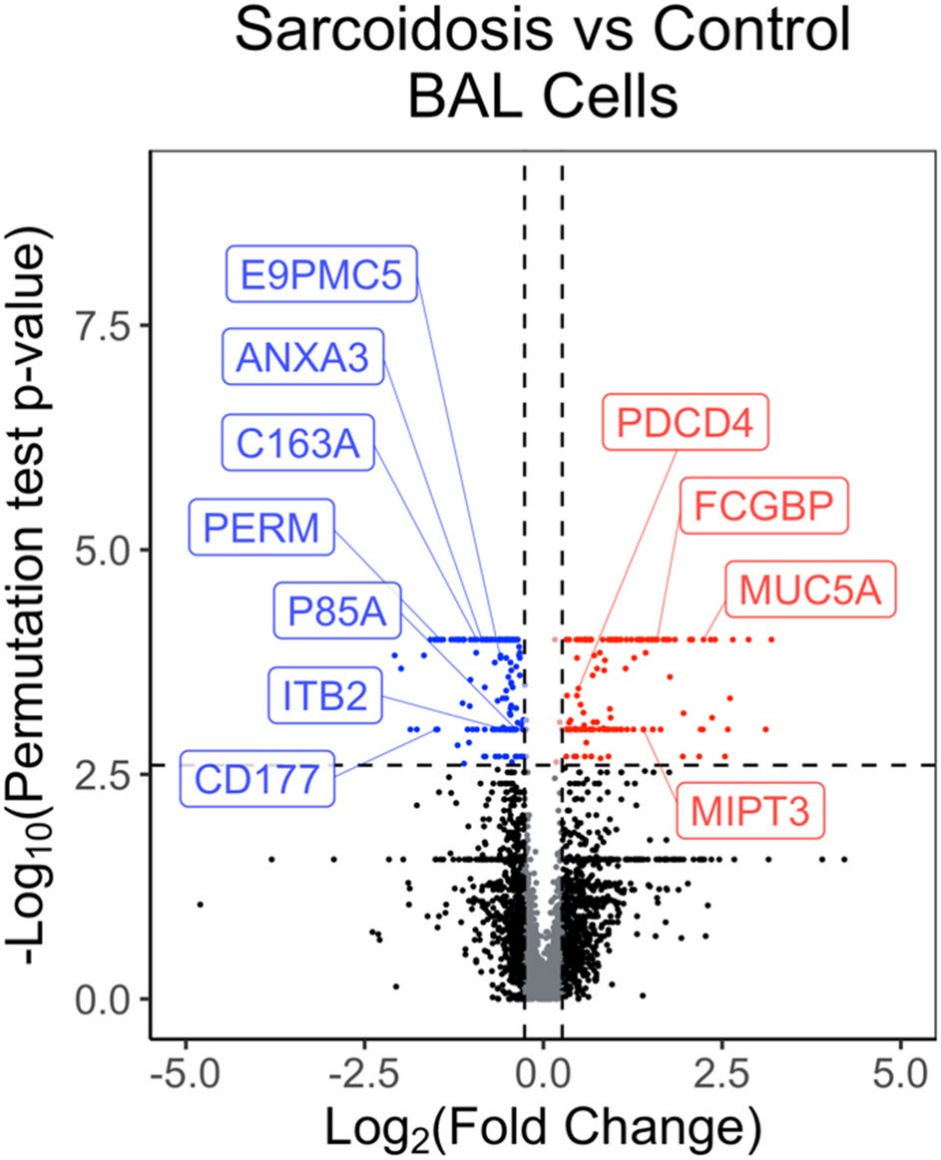
Volcano plot showing the differentially expressed BAL cell proteins. An individual dot represents each protein. The log_2_ fold change is plotted on the x-axis, and the log_2_ FDR corrected p-value is plotted on the y-axis. The horizontal dashed line corresponds to statistical significance from the permutation test (B and H corrected p-value = 0.0025) on a numerical scale, and the vertical line corresponds to a 1.2-fold change. The protein depicted by red dots are more abundant in sarcoidosis and the ones in blue dots are more abundant in controls. The black dots indicate the proteins that do not show a statistically significant change. *MUC5A *Mucin 5A,* FCGBP *IgG Fc-binding protein, * MIPT3 *TRAF3-interacting protein (also called Interleukin-13 receptor alpha 1-binding protein), *PDCD4 *Programmed cell death protein 4,* P85A *Phosphatidylinositol 3-kinase regulatory subunit alpha,* ITB2 * Integrin beta-2,* E9PMC5 *T cell immune regulator 1,* ANXA3 *Annexin A3, *CD163 *Scavenger receptor cysteine-rich type 1 protein,* CD177 *CD177 antigen,* PERM* Myeloperoxidase*.*

We used a stringent permutation testing and identified 272 differentially expressed proteins controlling for an FDR of ≤ 5%, Fig. 1 (Supplemental Table S1; ‘DE Proteins’ tab) between cases and controls. Table 3 lists the differentially expressed proteins that showed the most significant changes. Several other proteins that were differentially expressed included myeloperoxidase, T-cell immune regulator, cathepsin G, integrin subunit beta_2_, integrin subunit alpha M, myosin light chain, matrix metalloproteinase 9, PI3K regulator subunit, APOE, interleukin-13 receptor alpha 1-binding protein (TRAF3-interacting protein 1), and SERPINA1.

**Table 3:** Top ten differentially expressed cellular proteins comparing controls with sarcoidosis.

Protein name	B and H corrected p-value	Log_2_ fold change
Mucin-5AC	< 0.0001	2.24
Glutamate-rich protein 3	< 0.0001	2.65
Long-chain-fatty-acid-CoA ligase 1	< 0.0001	− 0.97
Keratin, type I cytoskeletal 18	< 0.0001	1.46
Mucin-5B	< 0.0001	1.72
D-3-phosphoglycerate dehydrogenase	< 0.0001	− 1.48
PDZ and LIM domain protein 1	< 0.0001	1.39
Vinculin	< 0.0001	− 0.62
Methyl-CpG-binding protein 2	< 0.0001	1.34
Cluster of endoplasmin	< 0.0001	− 0.55

### Biological relevance of the differentially expressed proteins in the BAL cells of cases compared to controls

**Figure 2:**
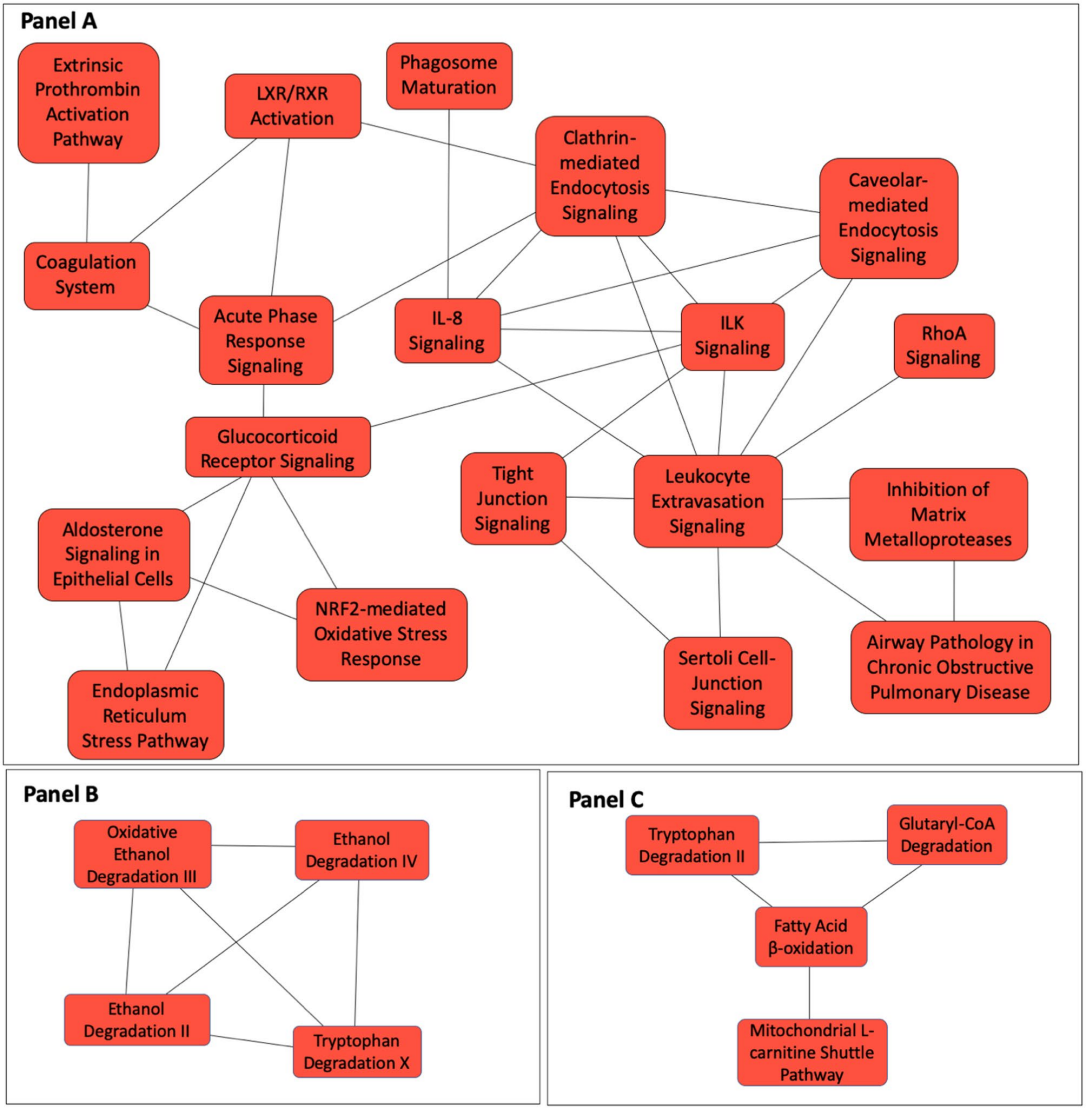
Cellular canonical pathways represented by differentially expressed proteins between sarcoidosis and controls implementing Overlapping Canoncial Pathway functionality in IPA. The 273 differentially expressed proteins map to thirty statistically significant canonical pathways. Each canonical pathway is represented as a node. The edges indicated at least two common proteins between the nodes to indicate shared biological function. Three clusters of overlapping pathways were identified. A larger cluster of overlapping canonical pathways includes diverse biological functions including IL-8, ILK, RhoA signaling, caveolin and clathrin-mediated endocytic signaling, NRF2-mediated oxidant response signaling and glucocorticoid receptor signaling (Panel **A**). The other two of have limited number of nodes and are involved in metabolic functions (Panels **B, C**).

To determine the biological significance of the differentially expressed proteins, we performed IPA core analysis to identify the canonical pathways that map to these proteins. The pathways that met the statistical threshold (− log[p-value] ≥ 1.3) and the proteins assigned to each canonical pathway are listed in Table 4. These include phagosome maturation, leukocyte extravasation signaling, tight junction signaling, ILK signaling, IL-8 signaling, clathrin-mediated endocytosis signaling, caveolin-mediated endocytosis signaling, glucocorticoid receptor signaling, NRF2-mediated oxidative stress response and RhoA signaling (Fig. 2). We also identified pathways linked to matrix turnover and glucocorticoid receptor signaling. Several metabolic pathways such as fatty acid β-oxidation, mitochondrial dysfunction, ethanol degradation, tryptophan metabolism and NRF2-mediated oxidant response also differed between controls and sarcoidosis subjects. The z-score indicating the activation state was available for fatty acid β-oxidation (− 2.5), leukocyte extravasation signaling (− 0.6), coagulation system (− 0.5), inhibition of matrix metalloproteases (1.0), ILK signaling (− 0.4), ethanol degradation (− 1.0), IL-8 signaling (− 1.7) and acute phase response signaling (− 1.9).

**Table 4:** Canonical  pathways represented by cellular proteins differentially expressed between sarcoidosis and control subjects.

Ingenuity canonical pathway	log (B-H p-value)	Molecules	Protein names
Fatty acid β-oxidation I	3.1	HSD17B10, ACSL3, HSD17B4, HADHA, ACAA2, ACSL1	Hydroxysteroid 17-beta dehydrogenase 10, Acyl-CoA synthetase long chain family member 3, Hydroxysteroid 17-beta dehydrogenase 4, Hydroxyacyl-CoA dehydrogenase trifunctional multienzyme complex subunit alpha, Acetyl-CoA acyltransferase 2, Acyl-CoA synthetase long chain family member 1
Phagosome maturation	2.58	DYNC1H1, NSF, MPO, TCIRG1, ATP6V0D1, NCF2, RAB7A, CTSG, RILP, EEA1	Dynenin cytoplasmic 1 heavy chain 1, N-ethylmaleimide sensitive factor vesicle fusing ATPase, Myeloperoxidase, T cell immune regulator 1, ATPase hydrogen transporting unit v0 subunit d1, Neutrophil cytosolic factor 2, Rab interacting lysosomal protein, Cathepsin G, RAB71 member Ras oncogene family, early endosome antigen 1
Leukocyte extravasation signaling	2.22	ITGB2, ITGAM, MYL6, EZR, MMP8, PIK3R1, NCF2, CTNNA1, AFDN, VCL, MMP9	Integrin subunit beta 2, Integrin subunit alpha M, Myosin light chain 6, Ezrin, Matrix metallopeptidase 8, Phosphoinositide-3-kinase regulatory subunit 1, Neutrophil cytosolic factor 2, Catenin alpha 1, Afadin, Adherens junction formation factor, Vinculin, Matrix metallopeptidase 9
Coagulation system	2.22	F5, F13A1, SERPINA1, FGA, A2M	Coagulation factor V, Coagulation factor XIII A chain, Serpin family A member 1, Fibrinogen alpha chain, Alpha-2-macroglobulin
Sertoli cell-sertoli cell junction signaling	2.19	EPB41, TJP2, CGN, CTNNA1, SPTB, AFDN, SPTA1, VCL, SPTAN1, A2M	Erythrocyte membrane protein band 4.1, Tight junction protein 2, Cingulin, Catenin alpha 1, Spectrin beta, erythrocytic, Afamin, Adherens junction formation factor, Spectrin alpha, Erythrocytic 1, Vinculin, Spectrin alpha, Non-erythrocytic 1, Alpha-2-macroglobulin
Inhibition of matrix metalloproteases	2.19	HSPG2, ADAM17, MMP8, A2M, MMP9	Heparan sulfate proteoglycan 2, ADAM metallopeptidase domain 17, Matrix metallopeptidase 8, Alpha-2-macroglobulin, Matrix metallopeptidase 9
Tight junction signaling	1.92	EPB41, NSF, TJP2, MYL6, CGN, CTNNA1, AFDN, VCL, SPTAN1	Erythrocyte membrane protein band 4.1, N-ethylmaleimide sensitive factor, Vesicle fusing ATPase, Tight junction protein 2, Myosin light chain 6, Cingulin, Catenin alpha 1, Afadin, Adherens junction formation factor, Vinculin, Spectrin alpha, Non-erythrocytic 1
Glucocorticoid receptor signaling	1.74	HSP90B1, KRT8, PIK3R1, SLPI, KRT18, GTF2E2, KRT5, FKBP5, CD163, HSPA5, A2M, NR3C1, KRT4	Heat shock protein 90 beta family member 1, Deratin 8, Phosphoinositide-3-kinase regulatory subunit 1, Secretory leukocyte peptidase inhibitor, Keratin 18, General transcription factor IIE subunit 2, Keratin 5, FK506 binding protein 5, CD163 molecule, Heat shock protein family A (Hsp70) member 5, Alpha-2-macroglobulin, nuclear receptor subfamily 3 group C member 1, Keratin 4
ILK signaling	1.53	FLNB, ITGB2, MYL6, FLNA, PIK3R1, VIM, KRT18, VCL, MMP9	Filamin B, Integrin subunit beta 2, Myosin light chain 6, filamin A, Phosphoinositide-3-kinase regulatory subunit 1, Vimentin, Keratin 18, Vinculin, Matrix metallopeptidase 9
Extrinsic prothrombin activation pathway	1.53	F5, F13A1, FGA	Coagulation factor V, Coagulation factor XIII A chain, Fibrinogen alpha chain
Glutaryl-CoA degradation	1.53	HSD17B10, HSD17B4, HADHA	Hydroxysteroid 17-beta dehydrogenase 10, Hydroxysteroid 17-beta dehydrogenase 4, Hydroxyacyl-CoA dehydrogenase trifunctional multienzyme complex subunit alpha
Ethanol degradation II	1.53	ALDH4A1, AKR1A1, ALDH1A1, ACSL1	Aldehyde dehydrogenase 4 family member A1, Aldo–keto reductase family 1 member A1, Aldehyde dehydrogenase 1 family member A1, Acyl-CoA synthetase long chain family member 1
Mitochondrial L-carnitine shuttle pathway	1.52	ACSL3, CPT1A, ACSL1	Acyl-CoA synthetase long chain family member 3, Carnitine palmitoyltransferase 1A, Acyl-CoA synthetase long chain family member 1
Mitochondrial dysfunction	1.51	GSR, HSD17B10, CPT1A, ATP5PO, ACO2, COX5A, VDAC1, UQCRC1	Glutathione-disulfide reductase, Hydroxysteroid 17-beta dehydrogenase 10, Carnitine palmitoyltransferase 1A, ATP synthase peripheral stalk subunit OSCP, Aconitase 2, Cytochrome c oxidase subunit 5A, Voltage dependent anion channel 1, Ubiquinol-cytochrome c reductase core protein 1
IL-8 signaling	1.51	ITGB2, ITGAM, PLD3, MPO, PIK3R1, NCF2, MMP9, LASP1, AZU1	Integrin β 2, Integrin α M, Phospholipase D member 3, Myeloperoxidase, PI3K regulator subunit, Neutrophil cytosolic factor 2, Matrix metalloproteinase 9, LIM and SH3 protein 1, Azurocidin 1
Aldosterone signaling in epithelial cells	1.51	HSP90B1, PIK3R1, DNAJC13, DNAJC3, HSPA5, PI4KA, DNAJB13, AHCY	Heat shock protein 90 beta family member 1, Phosphoinositide-3-kinase regulatory subunit 1, DnaJ heat shock protein family (Hsp40) member C13, DnaJ heat shock protein family (Hsp40) member C3, Heat shock protein family A (Hsp70) member 5, Phosphatidylinositol 4-kinase alpha, DnaJ heat shock protein family (Hsp40) member B13, Adenosylhomocysteinase
Folate polyglutamylation	1.51	MTHFD1, SHMT2	Methylenetetrahydrofolate dehydrogenase (cyclohydrolase and formyltetrahydrofolate synthetase 1), Serine hydroxymethyltransferase 2
Acute phase response signaling	1.48	ALB, APCS, PIK3R1, SERPINA3, SERPINA1, FGA, NR3C1, A2M	Albumin, Amyloid P component (serum), Phosphoinositide-3-kinase regulatory subunit 1, Serpin family A member 3, Serpin family A member 1, Fibrinogen alpha chain, nuclear receptor subfamily 3 group C member 1, Alpha-2-macroglobulin
Caveolar-mediated endocytosis signaling	1.47	FLNB, ITGB2, ALB, ITGAM, FLNA	Filamin B, Integrin beta 2, Albumin, Integrin alpha M, Filamin A
Oxidative ethanol degradation III	1.46	ALDH4A1, ALDH1A1, ACSL1	Aldehyde dehydrogenase 4 family member A1, Aldehyde dehydrogenase 1 family member A1, Acyl-CoA synthetase long chain family member 1
Endoplasmic reticulum stress pathway	1.46	HSP90B1, DNAJC3, HSPA5	Heat shock protein 90 beta family member 1, DnaJ heat shock protein family (Hsp40) member C3, Heat shock protein family A (Hsp70) member 5
Superpathway of serine and glycine biosynthesis I	1.31	PHGDH, SHMT2	Phosphoglycerate dehydrogenase, Serine hydroxymethyltransferase 2
Tryptophan degradation X (mammalian, via tryptamine)	1.31	ALDH4A1, AKR1A1, ALDH1A1	Aldehyde dehydrogenase 4 family member A1, Aldo–keto reductase family 1 member A1, Aldehyde dehydrogenase 1 family member A1
Tryptophan degradation III (eukaryotic)	1.31	HSD17B10, HSD17B4, HADHA	Hydroxysteroid 17-beta dehydrogenase 10, Hydroxysteroid 17-beta dehydrogenase 4, Hydroxyacyl-CoA dehydrogenase trifunctional multienzyme complex subunit alpha
Ethanol degradation IV	1.31	ALDH4A1, ALDH1A1, ACSL1	Aldehyde dehydrogenase 4 family member A1, Aldehyde dehydrogenase 1 family member A1, Acyl-CoA synthetase long chain family member 1
NRF2-mediated oxidative stress response	1.3	GSR, AKR1A1, PIK3R1, DNAJC13, DNAJC3, FKBP5, DNAJB13, FTH1	Glutathione-disulfide reductase, Aldo–keto reductase family 1 member A1, Phosphoinositide-3-kinase regulatory subunit 1, DnaJ heat shock protein family (Hsp40) member C13, DnaJ heat shock protein family (Hsp40) member C3, FK506 binding protein 5, Ferritin heavy chain 1, DnaJ heat shock protein family (Hsp40) member B13
LXR/RXR activation	1.3	APOE, ALB, SERPINA1, FGA, MMP9, CLU	Apolipoprotein E, Albumin, serpin family A member 1, Fibrinogen alpha chain, Matrix metallopeptidase 9, Clusterin
Clathrin-mediated endocytosis signaling	1.3	APOE, ITGB2, ALB, PIK3R1, RAB7A, SERPINA1, SH3GLB2, CLU	Apoliprotein E, Integrin beta 2, Albumin, PI3K regulator subunit, RAB71 member Ras oncogene family, Serpin family member A1, SH3 domain containing GRB2 like endophilin B2, Clusterin
Airway pathology in chronic obstructive pulmonary disease	1.3	MMP8, MMP9	Matrix metallopeptidase 8, Matrix metallopeptidase 9
RhoA signaling	1.3	SEPT9, MYL6, EZR, BAIAP2, PI4KA, KTN1	Septin 9, Myosin light chain 6, Ezrin, BAI1 associated protein 2, Phosphatidylinositol 4-kinase alpha, Kinectin 1

### Differences in the bronchoalveolar lavage fluid proteins between sarcoidosis and controls and between sarcoidosis phenotypes

**Figure 3:**
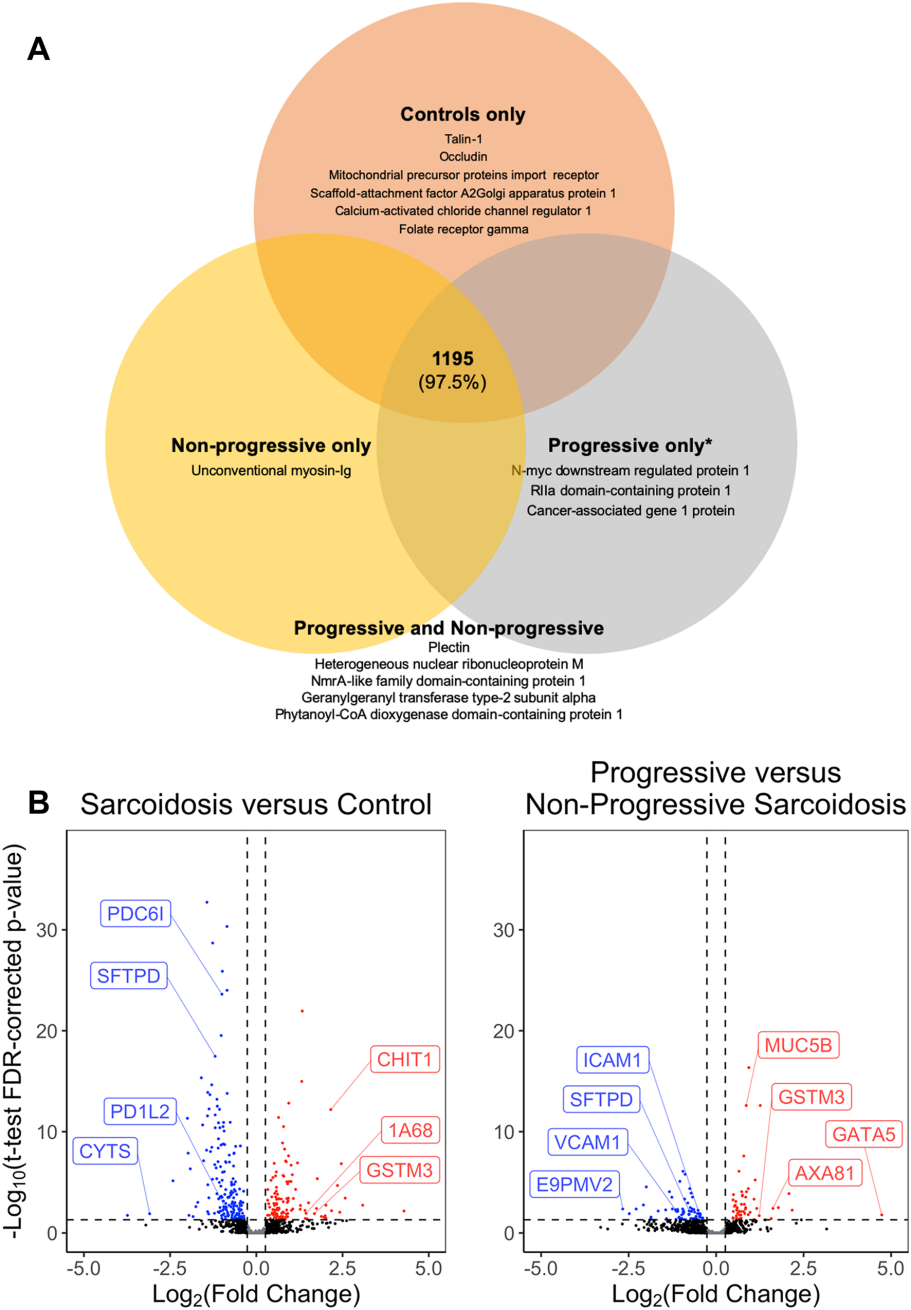
The BALF proteins detected in the controls and sarcoidosis cases. (**A**) The spectral database search identified 1,231 proteins of which 1,195 were detected in control, progressive and non-progressive subjects. Seven proteins were identified in control subjects but not in sarcoidosis cases. Five protein were present in sarcoidosis cases but not in controls, and four* proteins were detected in progressive sarcoidosis cases. (**B**) Volcano plot showing the differentially expressed BALF proteins. An individual dot represents each protein. The log_2_ fold change is plotted on the x-axis, and the log_2_ FDR corrected p-value is plotted on the y-axis. The horizontal dashed line corresponds to a corrected p-value = 0.05 on a numerical scale, and the vertical line corresponds to a 1.2-fold change. The left panel compares sarcoidosis to controls, and the right panel examines progressive and non-progressive subjects. The proteins depicted by red dots are more abundant in sarcoidosis (left panel), or progressive sarcoidosis (right panel) and have a positive log fold change. The blue dots are more abundant in controls (left panel) or non-progressive sarcoidosis (right panel). The black dots indicate proteins that do not show a statistically significant change. *CHIT1* Chitotriosidase, *GSTM3* Glutathione-*S*-transferase, *1A68* HLA class I histocompatibility antigen, *SFTPD* Pulmonary surfactant-associated protein D, *PDC61* Programmed cell death 6-interacting protein, *PD1L2* Programmed cell death 1 ligand 2, *HMGA1 * High mobility group protein HMG-I, *CYTS* Cystatin-S, *VCAM1* Vascular cell adhesion protein, *E9PMV2* HLA class II histocompatibility antigen, DQ alpha 1 chain, *ICAM1* Intercellular adhesion molecule 1, *AXA81* Annexin A8, *GATA5* Transcription factor GATA-5, *MUC5B* Mucin-5B. *One detected protein was an uncharacterized protein.

We examined BALF from seven control and ten sarcoidosis subjects. All BALF samples were analyzed by label-free mass spectrometry in triplicates. We identified 1,293 BALF proteins at an FDR of ≤ 1% (Supplemental Table S2; ‘Original File’ tab). These included 62 proteins that matched to the decoy (reverse) sequences or cRAP database such as keratins, filaggrin, cartilage matrix proteins, which were not considered for further analysis. The remaining 1,231 included 1,195 proteins present in all patients and controls. Seven proteins were only detected in controls and not in sarcoidosis cases, while five proteins were present only in sarcoidosis cases but not in control BALF. There were 12 proteins detected in controls and non-progressive cases but not in progressive sarcoidosis, five proteins in control and progressive cases but not in non-progressive sarcoidosis, one protein in only non-progressive but not in controls or progressive sarcoidosis, and four proteins were detected in only progressive but not in non-progressive sarcoidosis or controls (Fig. 3A). Peptides from the 1,231 BALF proteins (Supplemental Table S2; HAP CON REV tab) included proteins that originate from inflammatory cells and epithelial cell such as chitotriosidase-1, macrophage colony stimulating factor, Fc-gamma RIII-alpha, macrophage migration inhibitory factor (macrophage), human neutrophil defensin 3, neutrophil elastase (neutrophils), lymphocyte antigen, lymphocyte cytosolic protein (lymphocytes), aquaporin 1 and 5 (type 1 alveolar epithelial cells), and surfactant protein B (type 2 alveolar epithelial cells). Sixty-nine high abundance and immunoglobulin proteins or immunoglobulin fractions that were not completely removed by the high-abundance protein depletion column were also detected. These proteins were included for functional analysis as these proteins are crucial for many biological functions. Good quality quantitative spectral data was available to compare 1,223 of the 1,231 proteins in sarcoidosis vs. control subjects (Supplemental Table S3; ‘Sarc vs. control’ tab) and 1,206 of 1,231 proteins in progressive vs. non-progressive pulmonary sarcoidosis subjects (Supplemental Table S3; ‘P vs NP’ tab).

**Figure 4:**
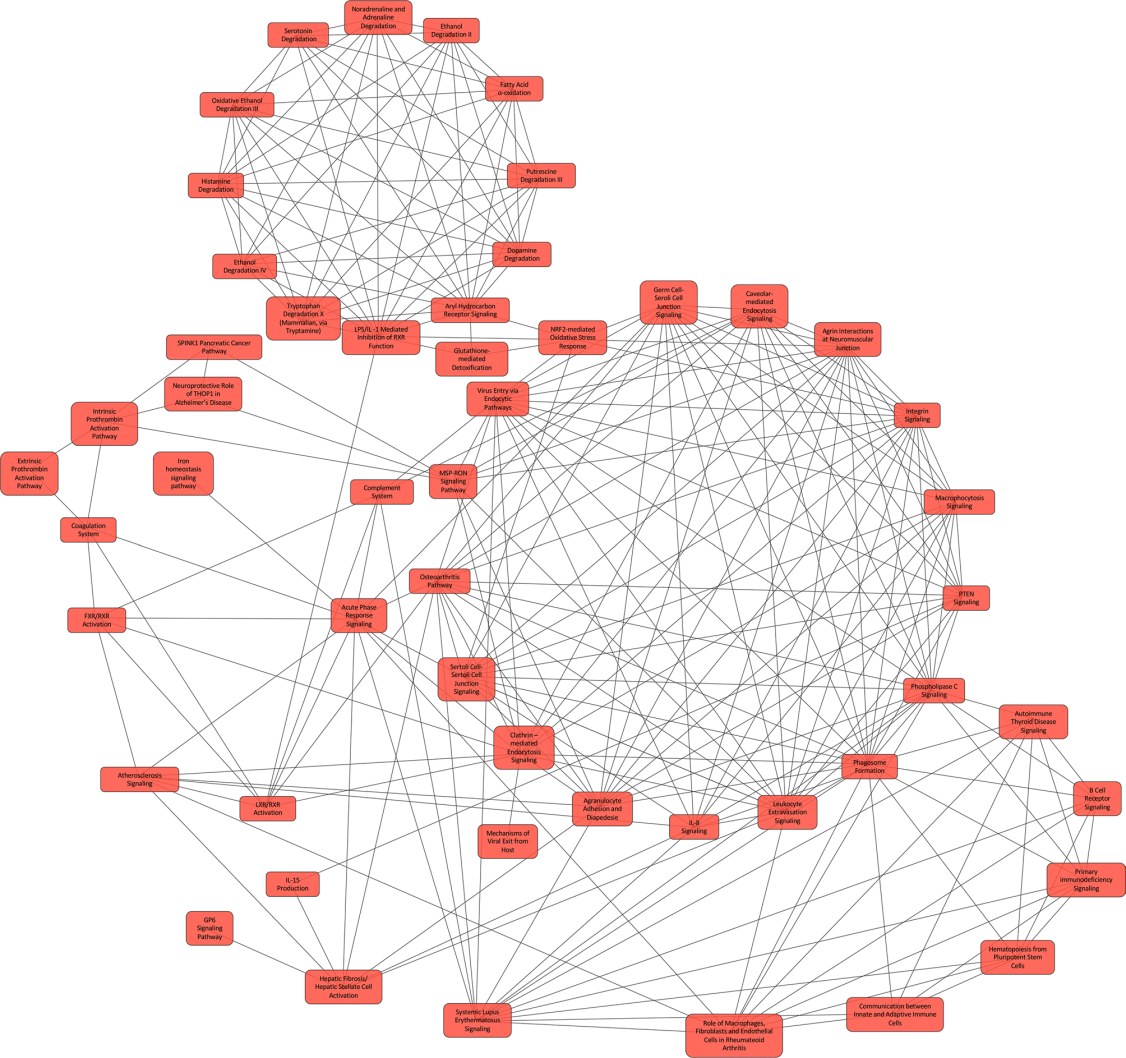
The canonical pathway represented by differentially expressed proteins in BALF between sarcoidosis and controls implementing Overlapping Canonical Pathway functionality in IPA. The 293 differentially expressed proteins map to 65 statistically significant canonical pathways. Each canonical pathway is represented as a node. The edges indicated at least two common proteins between the nodes to denote shared biological function. Complex network of pathways with diverse functions including immunological processes, signal transduction by kinases, acute phase response signaling, NRF2-mediated antioxidant response and several metabolic pathways were detected in this analysis.

We identified 293 differentially expressed proteins in sarcoidosis (n = 10) compared to the seven control subjects (Supplemental Table S3; Sarcoidosis vs Control tab), Fig. 3B. These proteins included chitotriosidase-1, serum amyloid protein P, surfactant protein D, S100P, inter-alpha-trypsin inhibitor, annexin, glutathione-*S*-transferase, interleukin-1 receptor accessory protein, cystatin-5, caveolin, choline transport protein, Fc-gamma RII-a, (Fcγ-binding protein), interleukin 6 receptor, programmed cell death 1 ligand 2, and aquaporin-1. The proteins with most significant differences with a higher abundance in sarcoidosis or controls are listed in Table 5. To find the biological relevance of the differentially expressed proteins, we determined the canonical pathways that map to these proteins (Table 6). These pathways include phagosome formation and maturation, IL-8 signaling, IL-12 signaling in macrophages, clathrin and caveolin endocytic signaling, LXR/RXR activation, B cell receptor signaling, communication between innate and adaptive immune cells, aryl hydrocarbon receptor signaling and NRF2-mediated oxidative stress response. Kinases signaling pathways such as PTEN, phospholipase C and GP6 signaling also map to the differentially expressed proteins. Overlapping Canonical Pathway analysis identified highly intricate network of pathways participating in immunological functions, acute phase response and metabolic processes (Fig. 4). The z-score indicating the activation state was available for LXR/RXR activation (2.9), acute phase response signaling (1.39), complement system (− 0.8), coagulation system (− 0.816), agrin interactions at the neuromuscular junction (− 1.633), glutathione-mediate detoxification (1.3), osteoarthritis pathways (− 0.4), SPINK1 pancreatic cancer pathway (1.6), intrinsic prothrombin activation pathway (− 0.5), phospholipase C signaling (− 0.6), serotonin degradation (− 1.3), BAG2 Signaling Pathway (− 1.), neuroprotective role of THOP1 in Alzheimer’s disease (− 2.2), leucocyte extravasation signaling (− 1.4), IL-8 signaling (− 0.4), GP6 signaling Pathway (0.8), PTEN signaling (2.5) and integrin signaling (− 1.9).

**Table 5:** Top differentially expressed BALF proteins in sarcoidosis vs controls.

Uniprot ID	Gene symbol	Protein name	Signal Log-ratio	p-value	p.fdr
**Proteins higher in sarcoidosis cases (compared to control subjects)**					
H3BNG3	H3BNG3	40S ribosomal protein S	4.28	1.13^–03^	6.86^–03^
M0QZ52	M0QZ52	Calmodulin	3.08	2.35^–04^	1.83^–03^
D6RE83	D6RE83	Ubiquitin carboxyl-terminal hydrolase	2.58	3.77^–05^	3.64^–04^
O60218	AK1BA	Aldo–Keto Reductase Family 1	2.46	7.39^–09^	1.46^–07^
O00764	PDXK	Pyridoxal Kinase	2.42	1.53^–03^	9.01^–03^
P02042	HBD	Hemoglobin Subunit Delta	2.35	1.62^–06^	2.09^–05^
P07585	PGS2	Decorin	2.18	1.04^–03^	6.39^–03^
Q13231	CHIT1	Chitotriosidase-1	2.16	1.18^–14^	6.59^–13^
P07451	CAH3	Carbonic anhydrase 3	2.02	7.55^–03^	3.38^–02^
Q6UWP8	SBSN	Suprabasin	2.00	4.08^–03^	2.00^–02^
**Proteins higher in controls subjects (compared to sarcoidosis cases)**					
Q53TN4	CYBR1	Cytochrome b reductase 1	− 1.75	3.45^–04^	2.53^–03^
H3BN27	H3BN27	Plasmolipin	− 1.83	5.08^–03^	2.41^–02^
C9JKI3	C9JKI3	Caveolin	− 1.92	2.60^–08^	4.68^–07^
K7EM38	K7EM38	Actin, cytoplasmic 2	− 1.95	3.70^–03^	1.84^–02^
A0A0C4DGI3	A0A0C4DGI3	Citrate synthase	− 1.98	5.31^–10^	1.33^–08^
P20142	PEPC	Gastricsin	− 2.00	1.02^–13^	4.82^–12^
P06870	KLK1	Kallikrein-1	− 2.42	4.84^–07^	7.14^–06^
P01036	CYTS	Cystatin 5	− 3.09	2.35^–03^	1.27^–02^
G3V1K2	G3V1K2	Ribitol-5-phosphate xylosyltransferase 1	− 3.74	3.70^–03^	1.84^–02^
P17096	HMGA1	High mobility group protein HMG-I	− 5.10	7.45^–04^	4.88^–03^

Fold changes calculated relative to controls resulting in positive log fold change if a protein was more abundant in sarcoidosis subjects and a negative log fold change when it was more abundant in controls.

*Signal Log-ratio *signal-log ratio (log_2_ magnitude of change), *p-value *protein level p-value calculated from beta distribution, *p.fdr *False discovery rate corrected p-value.

**Table 6:** Canonical pathways represented by proteins differentially expressed between sarcoidosis and control subjects in BALF.

Ingenuity canonical pathways	− log(B-H p-value)	Molecules	Protein name
Acute phase response signaling	10.1	A2M, APCS, APOA1, APOH, C3, C9, CP, FGB, FN1, HP, IL1RAP, IL6ST, ITIH2, ITIH3, ITIH4, RRAS, SERPINA1, SERPINA3, SERPINF1, SERPINF2	Alpha-2-macroglobulin, Serum amyloid P-component, Apolipoprotein A-I, Beta-2-glycoprotein 1, Complement C3, Complement component C9, Fibrinogen beta chain, Fibronectin, Haptoglobin, Interleukin-1 receptor accessory protein, Interleukin-6 receptor subunit beta, Inter-alpha-trypsin inhibitor heavy chain H2, Inter-alpha-trypsin inhibitor heavy chain H3, Inter-alpha-trypsin inhibitor heavy chain H4, Ras-related protein R-Ras, Alpha-1-antitrypsin, Alpha-1-antichymotrypsin, Pigment epithelium-derived factor, Alpha-2-antiplasmin
Complement system	8.26	C1QB, C1QC, C3, C6, C7, C9, CD55, CFD, CFH, ITGB2	Complement C1q subcomponent subunit B, Complement C1q subcomponent subunit C, Complement C3, Complement component C6, Complement component C7, Complement component C9, Complement decay-accelerating factor, Complement factor D, Complement factor H, Integrin beta-2
LXR/RXR activation	6.11	APOA1, APOA4, APOE, APOH, C3, C9, HADH, IL1RAP, ITIH4, S100A8, SERPINA1, SERPINF1, SERPINF2	Apolipoprotein A-I, Apolipoprotein A-IV, Apolipoprotein E, Beta-2-glycoprotein 1, Complement C3, Complement component C9, Hydroxyacyl-coenzyme A dehydrogenase (mitochondrial), Interleukin-1 receptor accessory protein, Inter-alpha-trypsin inhibitor heavy chain H4, Protein S100-A8, Alpha-1-antitrypsin, Pigment epithelium-derived factor, Alpha-2-antiplasmin
Coagulation system	3.43	A2M, FGB, PROS1, SERPINA1,SERPINC1,SERPINF2	Alpha-2-macroglobulin, Fibrinogen beta chain, Vitamin K-dependent protein S, Alpha-1-antitrypsin, SERPINC1, Alpha-2-antiplasmin
FXR/RXR activation	3.43	APOA1, APOA4, APOE, APOH, C3, C9, ITIH4, SERPINA1, SERPINF1, SERPINF2	Apolipoprotein A-I, Apolipoprotein A-IV, Apolipoprotein E, Beta-2-glycoprotein 1, Complement C3, Complement component C9, Inter-alpha-trypsin inhibitor heavy chain H4, Alpha-1-antitrypsin, Pigment epithelium-derived factor, Alpha-2-antiplasmin
Phagosome formation	3.43	FCGR2A, FN1, IGHG1, IGHG2, IGHG3, ITGA3, ITGB1, ITGB2, MRC1, RHOF	Low affinity immunoglobulin gamma Fc region receptor II-a, Fibronectin, Immunoglobulin heavy constant gamma 1, Immunoglobulin heavy constant gamma 2, Immunoglobulin heavy constant gamma 3, Integrin alpha-3, Integrin beta-1, Integrin beta-2, Macrophage mannose receptor 1, Rho-related GTP-binding protein
Agrin interactions at neuromuscular junction	3.38	ACTG1, AGRN, DAG1, ITGA3, ITGB1, ITGB2, LAMC1, RRAS	Actin (cytoplasmic 2), Agrin, Dystroglycan, Integrin alpha-3, Integrin beta-1, Integrin beta-2, Laminin subunit gamma-1, Ras-related protein R-Ras2
Caveolar-mediated endocytosis signaling	2.76	ACTG1, CAV1, CD55, HLA-A, ITGA3, ITGB1, ITGB2	Actin (cytoplasmic 2), Caveolin-1, Complement decay-accelerating factor, HLA class I histocompatibility antigen (A alpha chain), Integrin alpha-3, Integrin beta-1, Integrin beta-2
Clathrin-mediated endocytosis signaling	2.76	ACTG1, APOA1, APOA4, APOE, ITGB1, ITGB2, MET, S100A8, SERPINA1, TSG101, UBC	Actin (cytoplasmic 2), Apolipoprotein A-I, Apolipoprotein E, Integrin beta-1, Integrin beta-2, Hepatocyte growth factor receptor, Protein S100-A8, Alpha-1-antitrypsin, Tumor susceptibility gene 101 protein, Polyubiquitin-C
Primary immunodeficiency signaling	2.76	IGHD, IGHG1, IGHG2, IGHG3, IGHM, IGLL1, IGLL5	Immunoglobulin heavy constant delta, Immunoglobulin heavy constant gamma 1, Immunoglobulin heavy constant gamma 2, Immunoglobulin heavy constant gamma 3, Immunoglobulin heavy constant mu, Immunoglobulin lambda-like polypeptide 1, Immunoglobulin lambda-like polypeptide 1, Immunoglobulin lambda-like polypeptide 5
Glutathione-mediated detoxification	2.73	ANPEP, GSTA2, GSTM1, GSTM2, GSTM3	Aminopeptidase N, Glutathione-*S*-transferase A2, Glutathione-*S*-transferase Mu 1, Glutathione-*S*-transferase Mu 2, Glutathione-*S*-transferase Mu 3
Virus entry via endocytic pathways	2.64	ACTG1, CAV1, CD55, HLA-A, ITGA3, ITGB1, ITGB2, RRAS	Actin (cytoplasmic 2), Caveolin-1, Complement decay-accelerating factor, HLA class I histocompatibility antigen (A alpha chain), Integrin alpha-3, Integrin beta-1, Integrin beta-2, Ras-related protein R-Ras2
Iron homeostasis signaling pathway	2.64	ACO2, CD163, CP, CYBRD1, FTH1, HBD, HBG1, HP, LRP1	Aconitate hydratase (mitochondrial), Scavenger receptor cysteine-rich type 1 protein M130, Fibrinogen beta chain, Cytochrome b reductase 1, Ferritin heavy chain, Hemoglobin subunit delta, Hemoglobin subunit gamma-1, Haptoglobin, Prolow-density lipoprotein receptor-related protein 1
Osteoarthritis pathway	2.52	ALPG, ANXA2, DCN, FGFR3, FN1, IL1RAP, ITGA3, ITGB1, LRP1, S100A8, S100A9	Alkaline phosphatase (germ cell type), Annexin A2, Decorin, Fibroblast growth factor receptor 3, Fibronectin, Interleukin-1 receptor accessory protein, Integrin alpha-3, Integrin beta-1, Prolow-density lipoprotein receptor-related protein 1, Protein S100-A8, Protein S100-A9
SPINK1 pancreatic cancer pathway	2.49	CPM, CPN1, CPQ, CTSB, KLK1, KLK11	Carboxypeptidase M, Carboxypeptidase N catalytic chain, Carboxypeptidase Q, Cathepsin B, Kallikrein-1, Kallikrein-11
Autophagy	2.48	CTSB, CTSC, CTSD, CTSH, CTSS, CTSZ	Cathepsin B, Dipeptidyl peptidase 1, Cathepsin D, Pro-cathepsin H, Cathepsin S, Cathepsin Z
Hepatic fibrosis/hepatic stellate cell activation	2.44	A2M, COL5A1, COL6A1, COL6A3, FGFR2, FN1, ICAM1, IL1RAP, MET, VCAM1	Alpha-2-macroglobulin, Collagen alpha-1(V) chain, Collagen alpha-1(VI) chain, Collagen alpha-3(VI) chain, Fibroblast growth factor receptor 2, Fibronectin, Intercellular adhesion molecule 1, Interleukin-1 receptor accessory protein, Hepatocyte growth factor receptor, Vascular cell adhesion protein 1
Adenine and adenosine salvage	2.44	APRT, PNP	Adenine phosphoribosyltransferase, Purine nucleoside phosphorylase
Mechanisms of viral exit from host cells	2.44	ACTG1, CHMP2A, PDCD6IP, TSG101, VPS4A	Actin (cytoplasmic 2), Charged multivesicular body protein 2a, Programmed cell death 6-interacting protein, Tumor susceptibility gene 101 protein, Vacuolar protein sorting-associated protein 4A
Systemic lupus erythematosus signaling	2.42	C6, C7, C9, FCGR2A, HLA-A, IGHG1, IGHG2, IGHG3, IGHM, KLK1, RRAS	Complement component C6, Complement component C7, Complement component C9, Low affinity immunoglobulin gamma Fc region receptor II-a, HLA class I histocompatibility antigen (A alpha chain), Immunoglobulin heavy constant gamma 1, Immunoglobulin heavy constant gamma 2, Immunoglobulin heavy constant gamma 3, Immunoglobulin heavy constant mu, Kallikrein-1, Ras-related protein R-Ras2
Atherosclerosis signaling	2.42	APOA1, APOA4, APOE, ICAM1, ITGB2, S100A8, SERPINA1, VCAM1	Apolipoprotein A-I, Apolipoprotein A-IV, Apolipoprotein E, Intercellular adhesion molecule 1, Integrin beta-2, Protein S100-A8, Alpha-1-antitrypsin, Vascular cell adhesion protein 1
Intrinsic prothrombin activation pathway	2.42	FGB, KLK1, KLK11, PROS1, SERPINC1	Fibrinogen beta chain, Kallikrein-1, Kallikrein-11, Vitamin K-dependent protein S, Antithrombin-III
Tryptophan degradation × (Mammalian, via Tryptamine)	2.32	AKR1B10, ALDH2, ALDH3B1, ALDH7A1	Aldo–keto reductase family 1 member B10, Aldehyde dehydrogenase (mitochondrial), Aldehyde dehydrogenase family 3 member B1, Alpha-aminoadipic semialdehyde dehydrogenase
Hematopoiesis from pluripotent stem cells	2.17	IGHD, IGHG1, IGHG2, IGHG3, IGHM	Immunoglobulin heavy constant delta, Immunoglobulin heavy constant gamma 1, Immunoglobulin heavy constant gamma 2, Immunoglobulin heavy constant gamma 2, Immunoglobulin heavy constant gamma 3, Immunoglobulin heavy constant mu
S-adenosyl-l-methionine biosynthesis	2.11	MAT2A, MAT2B	*S*-adenosylmethionine synthase isoform type-2, Methionine adenosyltransferase 2 subunit beta
Aryl hydrocarbon receptor signaling	2.1	ALDH2, ALDH3B1, ALDH7A1, CTSD, GSTA2, GSTM1, GSTM2, GSTM3	Aldehyde dehydrogenase (mitochondrial), Aldehyde dehydrogenase family 3 member B1, Alpha-aminoadipic semialdehyde dehydrogenase, Cathepsin D, Glutathione-*S*-transferase A2, Glutathione-*S*-transferase Mu 1, Glutathione-*S*-transferase Mu 2, Glutathione-*S*-transferase Mu 3
Phospholipase C Signaling	2.1	CALM1 (includes others), FCGR2A, IGHG1, IGHG2, IGHG3, ITGA3, ITGB1, PLD3, PPP1CB, RHOF, RRAS	Calmodulin-1, Low affinity immunoglobulin gamma Fc region receptor II-a, Immunoglobulin heavy constant gamma 1, Immunoglobulin heavy constant gamma 2, Immunoglobulin heavy constant gamma 3, Integrin alpha-3, Integrin beta-1, Phospholipase D3, Serine/threonine-protein phosphatase PP1-beta catalytic subunit, Rho-related GTP-binding protein RhoF, Ras-related protein R-Ras2
LPS/IL-1 mediated inhibition of RXR function	2.01	ALDH2, ALDH3B1, ALDH7A1, APOE, FABP4, GSTA2, GSTM1, GSTM2, GSTM3, IL1RAP	Aldehyde dehydrogenase (mitochondrial), Aldehyde dehydrogenase family 3 member B1, Alpha-aminoadipic semialdehyde dehydrogenase, Apolipoprotein E, Fatty acid-binding protein, adipocyte, Glutathione-*S*-transferase A2, Glutathione- *S*-transferase Mu 1, Glutathione-*S*-transferase Mu 2, Glutathione-*S*-transferase Mu 3, Interleukin-1 receptor accessory protein
Ethanol degradation II	2	ADH1C, ALDH2, ALDH3B1, ALDH7A1	Alcohol dehydrogenase 1C, Aldehyde dehydrogenase (mitochondrial), Aldehyde dehydrogenase family 3 member B1, Alpha-aminoadipic semialdehyde dehydrogenase
Phagosome maturation	2	CTSB, CTSC, CTSD, CTSH,CTSS, CTSZ, HLA-A, TSG101	Cathepsin B, Dipeptidyl peptidase 1, Cathepsin D, Pro-cathepsin H, Cathepsin S, Cathepsin Z, HLA class I histocompatibility antigen (A alpha chain), Tumor susceptibility gene 101 protein
Extrinsic prothrombin activation pathway	1.89	FGB, PROS1, SERPINC1	Fibrinogen beta chain, Vitamin K-dependent protein S, Antithrombin-III
Noradrenaline and adrenaline degradation	1.89	ADH1C, ALDH2, ALDH3B1, ALDH7A1	Alcohol dehydrogenase 1C, Aldehyde dehydrogenase (mitochondrial), Aldehyde dehydrogenase family 3 member B1, Alpha-aminoadipic semialdehyde dehydrogenase
Histamine degradation	1.82	ALDH2, ALDH3B1, ALDH7A1	Aldehyde dehydrogenase (mitochondrial), Aldehyde dehydrogenase family 3 member B1, Alpha-aminoadipic semialdehyde dehydrogenase
Communication between innate and adaptive immune cells	1.75	HLA-A, IGHD, IGHG1, IGHG2, IGHG3, IGHM	HLA class I histocompatibility antigen (A alpha chain), Immunoglobulin heavy constant delta, Immunoglobulin heavy constant gamma 1, Immunoglobulin heavy constant gamma 2, Immunoglobulin heavy constant gamma 3, Immunoglobulin heavy constant mu
Germ cell-sertoli cell junction signaling	1.71	A2M, ACTG1, CDH1, ITGA3, ITGB1, NECTIN2, RHOF, RRAS	Alpha-2-macroglobulin, Actin (cytoplasmic 2), Cadherin-1, Integrin alpha-3, Integrin beta-1, Nectin-2, Rho-related GTP-binding protein RhoF, Ras-related protein R-Ras2
serotonin degradation	1.71	ADH1C, ALDH2, ALDH3B1, ALDH7A1, B4GAT1	Alcohol dehydrogenase 1C, Aldehyde dehydrogenase (mitochondrial), Aldehyde dehydrogenase family 3 member B1, Alpha-aminoadipic semialdehyde dehydrogenase, Beta-1,4-glucuronyltransferase 1
Oxidative ethanol degradation III	1.71	ALDH2, ALDH3B1, ALDH7A1	Aldehyde dehydrogenase (mitochondrial), Aldehyde dehydrogenase family 3 member B1, Alpha-aminoadipic semialdehyde dehydrogenase
Fatty acid α-oxidation	1.67	ALDH2, ALDH3B1, ALDH7A1	Aldehyde dehydrogenase (mitochondrial), Aldehyde dehydrogenase family 3 member B1, Alpha-aminoadipic semialdehyde dehydrogenase
Pyruvate fermentation to lactate	1.64	LDHA, LDHB	L-lactate dehydrogenase A chain, L-lactate dehydrogenase B chain
Glycogen biosynthesis II (from UDP-d-glucose)	1.64	GBE1, UGP2	1,4-alpha-glucan-branching enzyme, UTP–glucose-1-phosphate uridylyltransferase
Putrescine degradation iii	1.64	ALDH2, ALDH3B1, ALDH7A1	Aldehyde dehydrogenase (mitochondrial), Aldehyde dehydrogenase family 3 member B1, Alpha-aminoadipic semialdehyde dehydrogenase
BAG2 signaling pathway	1.64	ANXA2, CTSB,HSPA4, HSPA5	Annexin A2, Cathepsin B, Protein SPA1-RELATED 4, Endoplasmic reticulum chaperone BiP
Role of macrophages, fibroblasts and endothelial cells in rheumatoid arthritis	1.63	CALM1 (includes others), FN1, ICAM1, IGHG1, IGHG2, IGHG3, IL1RAP, IL6ST, LRP1, RRAS, VCAM1	Calmodulin-1, Low affinity immunoglobulin gamma Fc region receptor II-a, Fibronectin, Intercellular adhesion molecule 1, Immunoglobulin heavy constant gamma 1, Immunoglobulin heavy constant gamma 3, Interleukin-1 receptor accessory protein, Interleukin-6 receptor subunit beta, Prolow-density lipoprotein receptor-related protein 1, Ras-related protein R-Ras2, Vascular cell adhesion protein 1
B cell receptor signaling	1.59	CALM1 (includes others), FCGR2A, IGHD, IGHG1, IGHG2, IGHG3, IGHM, RRAS	Calmodulin-1, Low affinity immunoglobulin gamma Fc region receptor II-a, Low affinity immunoglobulin gamma Fc region receptor II-a, Immunoglobulin heavy constant gamma 1, Immunoglobulin heavy constant gamma 2, Immunoglobulin heavy constant gamma 3, Immunoglobulin heavy constant mu, Ras-related protein R-Ras2
Sertoli cell-sertoli cell junction signaling	1.59	A2M, ACTG1, CDH1, F11R, ITGA3, ITGB1, NECTIN2, RRAS	Alpha-2-macroglobulin, Actin (cytoplasmic 2), Cadherin-1, Junctional adhesion molecule A, Integrin alpha-3, Integrin beta-1, Nectin-2, Ras-related protein R-Ras2
Macropinocytosis signaling	1.59	ITGB1, ITGB2, MET, MRC1, RRAS	Integrin beta-1, Integrin beta-2, Hepatocyte growth factor receptor, Macrophage mannose receptor 1, Ras-related protein R-Ras2
Ethanol degradation IV	1.58	ALDH2, ALDH3B1, ALDH7A1	Aldehyde dehydrogenase (mitochondrial), Aldehyde dehydrogenase family 3 member B1, Alpha-aminoadipic semialdehyde dehydrogenase
NRF2-mediated oxidative stress response	1.57	ACTG1, FTH1, GSTA2, GSTM1, GSTM2, GSTM3, RRAS, USP14	Actin (cytoplasmic 2), Ferritin heavy chain, Glutathione-*S*-transferase A2, Glutathione-* S*-transferase Mu 1, Glutathione S-transferase Mu 2, Glutathione-*S*-transferase Mu 3, Ras-related protein R-Ras2, Ubiquitin carboxyl-terminal hydrolase 14
Aspartate degradation II	1.57	GOT2, MDH2	Aspartate aminotransferase (mitochondrial), Malate dehydrogenase (mitochondrial)
TCA cycle II (eukaryotic)	1.56	ACO2, CS, MDH2	Aconitate hydratase (mitochondrial), Citrate synthase (mitochondrial), Malate dehydrogenase (mitochondrial)
Agranulocyte adhesion and diapedesis	1.56	ACTG1, FN1, ICAM1, ITGA3, ITGB1, ITGB2, PECAM1, VCAM1	Actin (cytoplasmic 2), Fibronectin, Intercellular adhesion molecule 1, Integrin alpha-3, Integrin beta-1, Integrin beta-2, Platelet endothelial cell adhesion molecule, Vascular cell adhesion protein 1
Autoimmune thyroid disease signaling	1.56	HLA-A, IGHG1, IGHG2, IGHG3	HLA class I histocompatibility antigen (A alpha chain), Immunoglobulin heavy constant gamma 1, Immunoglobulin heavy constant gamma 2, Immunoglobulin heavy constant gamma 3
Neuroprotective role of THOP1 in Alzheimer's disease	1.53	CFD, HLA-A, KLK1, KLK11, PRSS8, SERPINA3	Complement factor D, HLA class I histocompatibility antigen (A alpha chain), Kallikrein-1, Kallikrein-11, Prostasin, Alpha-1-antichymotrypsin
Leukocyte extravasation signaling	1.51	ACTG1, F11R, ICAM1, ITGA3, ITGB1, ITGB2, PECAM1, VCAM1	Actin (cytoplasmic 2), Junctional adhesion molecule A, Intercellular adhesion molecule 1, Integrin alpha-3, Integrin beta-1, Integrin beta-2, Platelet endothelial cell adhesion molecule, Vascular cell adhesion protein 1
IL-8 signaling	1.51	CDH1, ICAM1, ITGB2, LASP1, PLD3, RHOF, RRAS, VCAM1	Cadherin-1, Intercellular adhesion molecule 1, Integrin beta-2, LIM and SH3 domain protein 1, Ras-related protein R-Ras2, Vascular cell adhesion protein 1
Glycolysis I	1.51	GAPDH, GPI, PFKL	Glyceraldehyde-3-phosphate dehydrogenase, Glucose-6-phosphate isomerase, ATP-dependent 6-phosphofructokinase (liver type)
Gluconeogenesis I	1.51	GAPDH, GPI, MDH2	Glyceraldehyde-3-phosphate dehydrogenase, Glucose-6-phosphate isomerase, Malate dehydrogenase (mitochondrial)
GP6 signaling pathway	1.51	CALM1 (includes others), COL5A1, COL6A1, COL6A3, FGB, LAMC1	Calmodulin-1, Low affinity immunoglobulin gamma Fc region receptor II-a, Collagen alpha-1(V) chain, Collagen alpha-1(VI) chain, Collagen alpha-3(VI) chain, Fibrinogen beta chain, Laminin subunit gamma-1
IL-15 production	1.49	DDR1, EPHB4, FGFR2, FGFR3, MET, ROS1	Epithelial discoidin domain-containing receptor 1, Ephrin type-B receptor 4, Fibroblast growth factor receptor 2, Fibroblast growth factor receptor 3, Hepatocyte growth factor receptor, Proto-oncogene tyrosine-protein kinase ROS
UDP-*N*-acetyl-d-galactosamine biosynthesis II	1.43	GPI, PGM3	Glucose-6-phosphate isomerase, Phosphoacetylglucosamine mutase
PTEN signaling	1.42	FGFR2, FGFR3, IGF2R, ITGA3, ITGB1, RRAS	Fibroblast growth factor receptor 2, Fibroblast growth factor receptor 3, Cation-independent mannose-6-phosphate receptor, Integrin alpha-3, Integrin beta-1, Ras-related protein R-Ras2
MSP-RON signaling pathway	1.38	ACTG1, ITGB2, KLK1, KLK11	Actin (cytoplasmic 2), Integrin beta-2, Kallikrein-1, Kallikrein-11
Dopamine degradation	1.38	ALDH2, ALDH3B1, ALDH7A1	Aldehyde dehydrogenase (mitochondrial), Aldehyde dehydrogenase family 3 member B1, Alpha-aminoadipic semialdehyde dehydrogenase
Integrin signaling	1.38	ACTG1, CAV1, ITGA3, ITGB1, ITGB2, PPP1CB, RHOF, RRAS	Actin (cytoplasmic 2), Caveolin-1, Integrin alpha-3, Integrin beta-1, Integrin beta-2, Serine/threonine-protein phosphatase PP1-beta catalytic subunit, Rho-related GTP-binding protein RhoF, Ras-related protein R-Ras2

**Figure 5:**
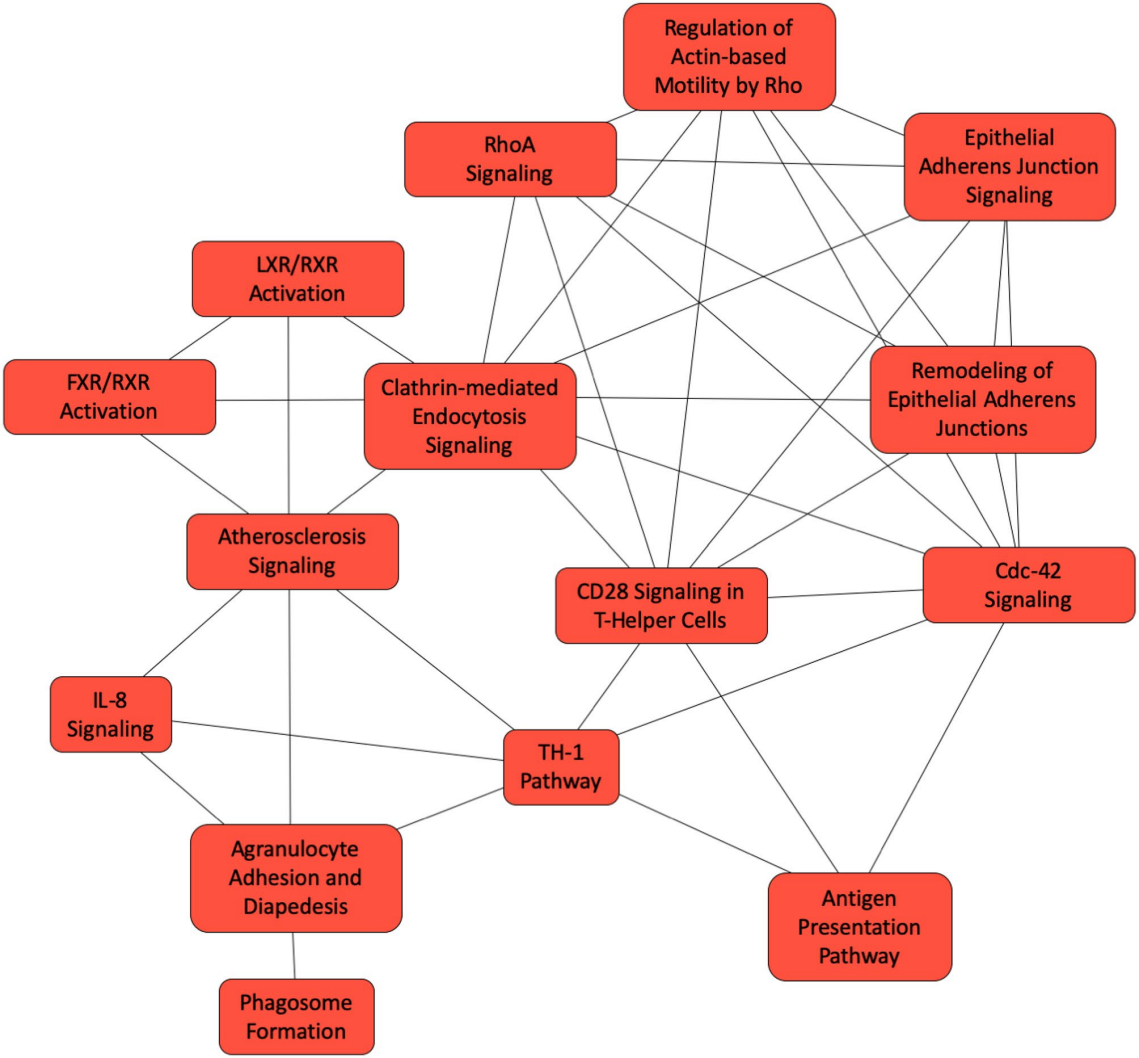
The canonical pathway represented by differentially expressed proteins in the BALF between progressive and non-progressive sarcoidosis implementing Overlapping Canonical Pathway functionality in IPA. The 121 differentially expressed proteins map to twenty-seven canonical pathways. Each canonical pathway is represented as a node. The edges indicated at least two proteins between the nodes to indicate shared biological function. The Th1 pathway, CD28 signaling, CDC-42 signaling and IL-8 signaling are highly-connected nodes detected with this analysis.

When we compared the BALF proteins between progressive vs. non-progressive sarcoidosis subjects (n = 5 each), there were 121 differentially expressed proteins. The proteins that differed between phenotypes included heat shock protein 90, glutathione-*S*-transferase, mucin-5B, annexin, CD5 antigen like protein (apoptosis inhibitor expressed by macrophages), chitotriosidase 1, ICAM 1, tropomyosin, integrin beta-2, pulmonary surfactant protein B and D, fatty acid binding protein, and HLA class II histocompatibility antigen DQ-α. The proteins with most significant differences with a higher abundance in cases with progressive disease compared to non-progressive disease are listed in Table 7. To determine the pathways that may contribute to the progression of sarcoidosis, we mapped the differentially expressed proteins between the progressive and non-progressive cases to canonical pathways in IPA (Table 8); these include aryl hydrocarbon receptor signaling, clathrin-mediated endocytic signaling, glutathione redox reaction, glutathione-mediated detoxification, antigen presentation pathway, phagosome formation, CD28 signaling in T-helper cells, CDC-42 signaling, RhoA signaling and PFKFB4 signaling pathway (Fig. 5). The z-score indicating the activation state was available for glycolysis (1.0), LXR/RXR (− 1.6) and IL-8 signaling (1.3).

**Table 7:** Top differential expressed BALF proteins comparing progressive to non-progressive cases.

Uniprot ID	Gene symbol	Protein name	Signal log-ratio	p-value	p.fdr
**Proteins higher in progressive sarcoidosis (compared to non-progressive)**					
Q9BWX5	GATA5	Transcription factor GATA-5	4.73	1.16^–03^	1.64^–02^
Q96DG6	CMBL	Carboxymethylenebutenolidase homolog	2.17	2.39^–04^	5.55^–03^
O00764	PDXK	Pyridoxal kinase	2.08	1.83^–06^	1.30^–04^
P68871	HBB	Hemoglobin subunit beta	1.78	9.67^–05^	3.15^–03^
Q5VT79	AXA81	Annexin A8-like protein 1	1.62	1.20^–04^	3.79^–03^
P69905	HBA	Hemoglobin subunit alpha	1.58	3.21^–03^	3.72^–02^
P12429	ANXA3	Annexin A3	1.26	6.35^–16^	2.55^–13^
P21266	GSTM3	Glutathione-*S*-transferase Mu 3	1.23	1.43^–03^	1.96^–02^
P08263	GSTA1	Glutathione-*S*-transferase A1	1.16	1.23^–04^	3.79^–03^
Q6P5S2	LEG1H	Protein LEG1 homolog	1.12	3.88^–08^	5.84^–06^
**Proteins higher in non-progressive sarcoidosis (compared to progressive)**					
P11047	LAMC1	Laminin subunit gamma-1	− 1.42	2.21^–04^	5.23^–03^
F8VR50	F8VR50	Actin-related protein 2/3complex subunit 3	− 1.44	2.21^–04^	5.23^–03^
P21695	GPDA	Glycerol-3-phosphate dehydrogenase	− 1.66	3.27^–04^	6.79^–03^
Q96C19	EFHD2	EF-hand domain-containing protein D2	− 1.86	4.01^–03^	4.32^–02^
P30464	1B15	HLA class I histocompatibility antigen, B alpha chain	− 1.86	2.10^–03^	2.72^–02^
O43866	CD5L	CD5 antigen-like	− 2.00	2.36^–07^	2.84^–05^
P31025	LCN1	Lipocalin-1	− 2.08	4.42^–05^	1.72^–03^
S4R3A2	S4R3A2	Fatty acid-binding protein	− 2.29	1.55^–04^	4.30^–03^
P23246	SFPQ	Splicing factor, proline- and glutamine-rich	− 2.48	7.88^–04^	1.23^–02^
E9PMV2	E9PMV2	HLA class II histocompatibility antigen, DQ alpha 1 chain	− 2.67	1.61^–04^	4.31^–03^

Fold changes calculated relative to non-progressive sarcoidosis cases resulting in a positive log fold change for proteins higher in progressive sarcoidosis and a negative log fold change for proteins higher in non-progressive sarcoidosis cases.

*Signal Log-ratio *signal-log ratio (log_2_ magnitude of change), *p-value *protein level p-value calculated from beta distribution, *p.fdr *false discovery rate corrected p-value.

**Table 8:** Canonical pathways mapping to proteins differentially expressed between progressive and non-progressive sarcoidosis.

Ingenuity canonical pathways	-log (B-H p-value)	Molecules	Protein names
Atherosclerosis signaling	4.05	APOA4, CLU, ICAM1, ITGB2, LCAT, LYZ, PON1, VCAM1	Apolipoprotein A-IV, Clusterin, Intercellular adhesion molecule 1, Integrin beta-2, Phosphatidylcholine-sterol acyltransferase, Lysozyme C, Serum paraoxonase/arylesterase 1, Vascular cell adhesion protein 1
Aryl hydrocarbon receptor signaling	3.11	ALDH9A1, BAX, GSTA1, GSTM3, GSTP1, HSP90AB1, HSPB1	4-trimethylaminobutyraldehyde dehydrogenase, Apoptosis regulator BAX, Glutathione-*S*-transferase A1, Glutathione-*S*-transferase Mu 3, Glutathione-*S*-transferase P, Heat shock protein HSP 90-beta, Heat shock protein beta-1
Clathrin-mediated endocytosis signaling	3.11	APOA4, ARPC3, ARPC4, CLU, ITGB2, LYZ, PON1, TFRC	Apolipoprotein A-IV, Actin-related protein 2/3 complex subunit 3, Actin-related protein 2/3 complex subunit 4, Clusterin, Integrin beta-2, Lysozyme C, Serum paraoxonase/arylesterase 1, Transferrin receptor protein 1
Glycolysis I	3.11	ENO2, GPI, PKM, TPI1	Gamma-enolase, Glucose-6-phosphate isomerase, Pyruvate kinase PKM, Triosephosphate isomerase
l-Cysteine degradation III	2.82	GOT1, MPST	Aspartate aminotransferase (cytoplasmic), 3-mercaptopyruvate sulfurtransferase
LXR/RXR activation	2.74	APOA4, CLU, LCAT, LYZ, PON1, VTN	Apolipoprotein A-IV, Clusterin, Phosphatidylcholine-sterol acyltransferase, Lysozyme C, Serum paraoxonase/arylesterase 1, Vitronectin
Complement System	2.74	C8A, CFH, CFI, ITGB2	Complement component C8, Complement factor H alpha chain, Integrin beta-2, Complement factor I, Intercellular adhesion molecule 1, Integrin beta-2
Glutathione redox reactions II	2.34	GLRX, PDIA3	Glutaredoxin-1, Protein disulfide-isomerase A3
Phenylalanine degradation I (aerobic)	2.34	PCBD1, QDPR	Pterin-4-alpha-carbinolamine dehydratase, Dihydropteridine reductase
Epithelial adherens junction signaling	2.34	ARPC3, ARPC4, BAIAP2, CDH1, JUP, TUBB4B	Actin-related protein 2/3 complex subunit 3, Actin-related protein 2/3 complex subunit 4, Brain-specific angiogenesis inhibitor 1-associated protein 2, Cadherin-1, Junction plakoglobin, Tubulin beta-4B chain
Gluconeogenesis	2.07	ENO2, GPI, MDH1	Gamma-enolase, Glucose-6-phosphate isomerase, Malate dehydrogenase, cytoplasmic
FXR/RXR activation	1.95	APOA4, CLU, LCAT, PON1, VTN	Apolipoprotein A-IV, Clusterin, Phosphatidylcholine-sterol acyltransferase, Serum paraoxonase/arylesterase 1, Vitronectin
Aspartate degradation II	1.95	GOT1, MDH1	Aspartate aminotransferase (cytoplasmic), Malate dehydrogenase, cytoplasmic
Remodeling of epithelial adherens junctions	1.95	ARPC3, ARPC4, CDH1, TUBB4B	Actin-related protein 2/3 complex subunit 3, Actin-related protein 2/3 complex subunit 4, Cadherin-1, Tubulin beta-4B chain
IL-8 signaling	1.93	BAX, CDH1, ICAM1, ITGB2, LASP1, VCAM1	Apoptosis regulator BAX, Cadherin-1, Intercellular adhesion molecule 1, Integrin beta-2, LIM and SH3, Vascular cell adhesion protein 1
Glutathione-mediated Detoxification	1.93	GSTA1, GSTM3, GSTP1	Glutathione-*S*-transferase A1, Glutathione-*S*-transferase Mu 3, Glutathione-*S*-transferase P
Antigen presentation pathway	1.73	HLA-B, HLA-DQA1, PDIA3	Protein disulfide-isomerase A3, HLA class II histocompatibility antigen (DQ alpha 1 chain), Protein disulfide-isomerase A3
Regulation of actin-based motility by Rho	1.62	ARPC3, ARPC4, BAIAP2, GSN	Actin-related protein 2/3 complex subunit 3, Actin-related protein 2/3 complex subunit 4, Brain-specific angiogenesis inhibitor 1-associated protein 2, Gelsolin
Glycogen degradation II	1.6	PGM1, TYMP	Phosphoglucomutase-1, Thymidine phosphorylase
PFKFB4 signaling pathway	1.6	GPI, HK3, TKT	Glucose-6-phosphate isomerase, Hexokinase-3, Transketolase
CDC-42 signaling	1.59	ARPC3, ARPC4, BAIAP2, HLA-B, HLA-DQA1	Actin-related protein 2/3 complex subunit 3, Actin-related protein 2/3 complex subunit 4, Brain-specific angiogenesis inhibitor 1-associated protein 2, Protein disulfide-isomerase A3, HLA class II histocompatibility antigen (DQ alpha 1 chain)
Glycogen degradation III	1.52	PGM1, TYMP	Phosphoglucomutase-1, Thymidine phosphorylase
Agranulocyte adhesion and diapedesis	1.37	FN1, ICAM1, ITGB2, PECAM1, VCAM1	Fibronectin, Intercellular adhesion molecule 1, Integrin beta-2, Platelet endothelial cell adhesion molecule, Vascular cell adhesion protein 1
CD28 signaling in T helper cells	1.36	ARPC3, ARPC4, HLA-B, HLA-DQA1	Actin-related protein 2/3 complex subunit 3, Actin-related protein 2/3 complex subunit 4, Protein disulfide-isomerase A3, HLA class II histocompatibility antigen (DQ alpha 1 chain)
RhoA signaling	1.36	ARPC3, ARPC4, BAIAP2, NRP2	Actin-related protein 2/3 complex subunit 3, Actin-related protein 2/3 complex subunit 4, Brain-specific angiogenesis inhibitor 1-associated protein 2, Neuropilin-2
Th1 pathway	1.36	HLA-B, HLA-DQA1, ICAM1, ITGB2	Protein disulfide-isomerase A3, HLA class II histocompatibility antigen (DQ alpha 1 chain), Intercellular adhesion molecule 1, Integrin beta-2
Phagosome formation	1.35	FN1, ITGB2, PDIA3, VTN	Fibronectin, Integrin beta-2, Protein disulfide-isomerase A3, Vitronectin
IL-12 signaling and production in macrophages	1.28	APOA4, CLU, LYZ, PON1	Apolipoprotein A-IV, Clusterin, Lysozyme C, Serum paraoxonase/arylesterase 1

## Discussion

Use of ‘omics’ tools to improve the understanding of sarcoidosis has been recognized as a high priority area of research in sarcoidosis^29^. We implemented an approach that coupled state-of-the-art mass spectrometry based proteomics with novel bioinformatics for a comprehensive characterization of the protein changes in the lung compartment in well-phenotyped cases. In the absence of well-characterized animal models, the examination of BAL cells provides an ex vivo model of the immune response in sarcoidosis. While proteomic studies have been conducted previously^23,26,28^, no prior study has comprehensively characterized mixed BAL cells and BALF. In addition, we established workflows for comprehensive characterization of BALF to obtain unprecedented coverage and detect proteins that originate from diverse cellular and extracellular sources. Ultimately, our approach to characterize mixed BAL cells captured the complex interplay between inflammatory cells in sarcoidosis. Specifically, in BAL cells and fluid we identified several pathways present in macrophages such as clathrin-mediated endocytic signaling and other phagocytic processes as well as redox-related pathways that were previously reported to be upregulated in sarcoidosis^23,30^. We also identified novel pathways implicated in sarcoidosis such as signaling by integrin-linked kinase, IL-8, and caveolar-mediated endocytic signaling in our studies comparing BAL cells from controls and sarcoidosis cases. The studies in BALF showed higher levels of chitotriosidase, a potential biomarker and an investigational agent for therapy^31,32^ when comparing cases to controls. Several of the biological pathways identified in the BAL cells were also identified in the BALF, suggesting that BALF is a useful biofluid to investigate mechanistic processes in sarcoidosis. In our comparison of cases with progressive vs. non-progressive sarcoidosis, we identified several novel pathways that may be involved in progression in sarcoidosis. These included CD28 signaling and PFKFB4 signaling. These results suggest that a systematic characterization of BALF may prove fruitful to develop disease models and classifiers with diagnostic and prognostic utility, while BALF and the cellular proteome will provide insight into the mechanisms underlying sarcoidosis as well as the processes that promote progressive disease.

We examined BAL cells as the inflammatory response is aberrant in sarcoidosis with (a) yet unknown antigen(s) triggering an exuberant although dysfunctional immune response with CD4 + T cells, Tregs, high levels of Th1 cytokines TNF-α, IFNγ, and IL-2^10,33,34^, along with inappropriate counter regulatory responses. Previous studies investigating protein changes in alveolar macrophages^23,26^ and gene expression changes in peripheral blood mononuclear cells^35^ found phagocytosis-related pathways to be upregulated in sarcoidosis subjects such as Fcγ receptor-mediated phagocytosis and clathrin-mediated endocytic signaling. We identified differences in cellular proteins mapping to phagosome maturation and clathrin-mediated endocytic signaling in sarcoidosis vs. controls BAL cells. Phagocytosis is crucial for innate and adaptive immune response and plays an essential role in antigen presentation, supporting the notion that sarcoidosis results from the response to an unknown external exposure requiring antigen processing and presentation for the development of disease. Similar to previous reports, we observed that the proteins involved in clathrin-mediated endocytic signaling differ in sarcoidosis cases when compared to controls. Additionally, caveolar-mediated endocytic signaling was also different between the two comparison groups. While both these pathways play a role in endocytic internalization of a variety of particles, again implicating exposure in disease ontogeny, these pathways also play a role in signal transduction and the regulation of many plasma membrane activities that have not been studied in sarcoidosis as well as have an influence on the immune response in alveolar macrophages^36,37^ and peripheral blood mononuclear cells^38^. In fact, the role of clathrin and caveolar pathways in the development of sarcoidosis has not been systematically studied. Thus, our findings suggest new pathways for investigation of potential disease pathogenesis and or cell regulation in sarcoidosis.

With an unbiased approach, we identified several canonical pathways mapping differentially expressed proteins that have not been previously linked to sarcoidosis, but would be likely to play a role in disease pathogenesis. These include integrin-linked kinase (ILK) signaling, IL-8 signaling, and inhibition of matrix metalloproteinases. ILK is an intracellular protein that primarily functions to connect integrins to the cytoskeletal proteins. The intracellular domain of ILK interacts with different proteins and regulates the phosphorylation of protein kinase B (PI3K)/AKT1 and glycogen synthase kinase 3B^39^. The downstream signaling cascade of PI3K/AKT activation includes activation of mTOR^25^, which is implicated in the development and the progression of sarcoidosis and has been proposed as a potential therapeutic target^40^. Thus, ILK-mediated mTOR activation could be a possible mechanism mediating inflammation in a subset of sarcoidosis cases. ILK signaling also activates c-Jun N-terminal kinase (JNK) via transcription-factor activator protein 1 (AP1) and regulates the gene expression of MMP9^41^ and also IL-8 signaling^42^. IL-8 is a chemokine in the CXC family and is produced by non-leucocytic and leucocytic cells including macrophages, and binds to CXCR1 and CXCR2 surface receptors^43^. Several cytokines such as TNF-α induce the production of IL-8^44^. Higher levels of IL-8 have been reported sarcoidosis BALF^45^ and serum, with the latter correlating with pulmonary^46^ and chronic disease^47^. IL-8 signaling has recently been reported to directly regulate adaptive T cell reactivity^48^ and phagosome function. Thus, our findings are not surprising but suggest that future studies investigating IL-8 signaling could improve the understanding of sarcoidosis pathogenesis and potentially phenotypes. They also highlight the importance of comprehensive characterization of the BAL cell protein changes in providing insight into sarcoidosis development and or progression, an approach that offers promise and is underutilized thus far in sarcoidosis research.

The examination of BALF revealed many proteins that are represented by canonical pathways that were also found in BAL cells. This indicates that biological mechanisms that contribute to the development of sarcoidosis can be identified in the BALF. When we compared BALF from sarcoidosis subjects to controls, similar to the findings from BAL cells, we identified several pathways that are linked to the inflammatory response. These included phagosome formation/maturation, clathrin- and caveolar-mediated endocytic signaling, LXR/RXR activation, IL-8 signaling, fatty acid oxidation, NRF2-mediated oxidative stress response and tryptophan degradation. Several of these pathways are also assigned to the proteins that are differentially expressed between progressive and non-progressive sarcoidosis cases. Some BALF pathways map to proteins that are only differentially expressed between progressive and non-progressive sarcoidosis. Specifically, we identified proteins mapping to CD28 signaling in T-helper cells, PFKFB4 (6-phosphofructo-2-kinase/fructose-2,6-biphosphatase 4) signaling and IL-12 signaling and production in macrophages. CD28 is a stimulatory immune checkpoint molecule of B7-CD28 superfamily with diverse roles in naïve and CD4 + T cells. The cytoplasmic tail of CD28 contains signaling motifs that are phosphorylated in response to TCR and CD28 stimulation^49^. Binding of the adaptor proteins to the activated motif, in turn phosphorylates and activates CDC-42^50^, culminating in the activation of JNK^51^. While we did not identify enrichment of canonical JNK pathways, BALF may only reflect some of the processes involved in sarcoidosis pathogenesis with secreted proteins. Regardless, the finding of differentially expressed BALF proteins mapping to CDC-42 and CD28 signaling suggests that they may possibly be involved in disease progression. Additionally, CD28 controls differentiation of Tregs from naïve CD4 T cells, providing novel mechanisms that may explain progression or remission of sarcoidosis. Interestingly, we identified PFKFB4 and IL-12 signaling also mapping to proteins that are differentially expressed in progressive vs. non-progressive cases. PFKFB4 is a bifunctional glycolytic enzyme that synthesizes and degrades fructose 2,6,-biphosphate. PFKFB4 regulates glucose metabolism and cell fate of dendritic cells^52^ and may provide a link for immunomodulatory effects by 1,25-dihydroxyvitamin D_3_ (1,25 (OH_2_) D_3_). Vanhewegan et al., identified PFKFB4 as a master regulator of 1,25 (OH)^2^ D_3_ induced DC tolerogenicity and inhibition of PFKFB4 signaling promotes secretion of proinflammatory cytokines including TNF-α^53^. The exact role of these pathways in the progression of pulmonary disease remains to be investigated, but our study suggests further investigation should be undertaken.

In pulmonary sarcoidosis, higher oxidant stress is reported in inflammatory cells in the lung^54^ and BALF^55^. In our study, the examination of mixed BAL cells indicated altration in redox balance in newly-diagnosed sarcoidosis subjects. Specifically, the mitochondrial l-carnitine shuttle pathway which is involved in fatty acid and lipid degradation, was mapped by proteins with differential abundance in controls compared to  sarcoidosis, suggesting that the mitochondrial metabolism is altered. Furthermore, we found differentially expressed proteins in pathways related to β-oxidation of fatty acids and mitochondrial dysfunction. We also identified several cytoprotective enzymes that mapped to NRF2 mediated oxidative stress response were differentially abundant in sarcoidosis compared to controls. NRF2 regulates mitochondrial redox homeostasis by several mechanisms such as detoxification of peroxides, regeneration of GSH, increased synthesis of GSH and NADPH and via the NRF2-Keap 1 response. Mitochondrial dysfunction occurs when the reactive oxygen species (ROS)-mediated stress overpowers the antioxidant defense system^56^. Bleomycin challenge in NRF2 knockout mice results in increased inflammatory makers, lower level of antioxidant enzymes, a bias towards Th2 response and increased fibrosis^57^. Taken together, these findings suggest that in sarcoidosis abnormal fatty acid and lipid degradation in the mitochondria cause the production of oxidants, with altered redox balance. It is possible that the detoxification mechanisms are overwhelmed causing mitochondrial dysfunction, production of reactive oxygen species that contribute to the inflammatory response seen in the lungs. NRF2 activators such as curcumin, sulforaphane, resveratrol, and quercetin counteract increased oxidant stress have a potential benefit in acute respiratory distress syndrome^58^, chronic obstructive pulmonary disease^59^, asthma^60^ and idiopathic pulmonary fibrosis^61^ and could be tested as a possible therapeutic strategy in sarcoidosis. The proteins differentially expressed between controls vs sarcoidosis and progressive vs. non-progressive sarcoidosis cases also mapped to Aryl hydrocarbon receptor signaling. AhR signaling is emerging as an important regulator of immunity in response to endogenous and exogenous ligands^62^ including tryptophan and serotonin metabolism. The differentially expressed proteins in both of the comparisons mapped to tryptophan /serotonin degradation but only reached statistical significance in the sarcoidosis vs control comparison. AhR signaling controls adaptive immunity by regulating T cell differentiation and by effecting antigen-presenting cells^63^. AhR regulates T cell response at multiple levels including T cell fate^64^. AhR is linked to induction of CD4 + Treg or Th17^65^ and Th22 cell differentiation directing the balance between effector and regulatory T cells. AhR signaling is implicated in other diseases with granulomatous inflammation. In Crohn’s disease, AhR RNA transcripts were markedly downregulated in the inflamed tissue and in the CD4 + T cells^66^. AhR signaling is also implicated in particulate induced granulomatous inflammation such as silicosis ^67^.

While we observe differences in the biological processes annotated to the differentially expressed proteins, systematic investigation of the BAL could provide the yet elusive biomarkers with diagnostic and prognostic value in sarcoidosis. In our dataset, we found higher BAL levels of chitotriosidase in sarcoidosis cases compared to controls. Chitotriosidase is a monocyte-macrophage-derived protein that is elevated in plasma and BALF and has been associated with sarcoidosis severity^68^. Another interesting protein with differential expression higher in the BALF in sarcoidosis compared to controls, programmed cell death 1 ligand 2 (PD-L2), is a ligand for programmed death-1 (PD1) receptor. PD-L2 is a transmembrane protein that is involved in immune checkpoint activity of PD1. In sarcoidosis, PD1 has been linked to the development of T cell exhaustion^69^ and a blockade of the PD1 pathway restored sarcoidosis CD4 proliferative capacity^70^. The notion that the PD1 pathway is involved in sarcoidosis is also strengthened by reports of sarcoidosis-like illness in patients receiving PD1 immune checkpoint modulators^71^. While the presence of individual proteins in our dataset is encouraging, we expect that the systematic examination of global protein changes in the BALF coupled with statistical approaches to construct a parsimonious model consisting of an orthogonal set of proteins will be the best approach for diagnosis and prognostication in sarcoidosis.

A network-based approach is a powerful framework for studying the organizational structure of complex systems^72^. Networks are represented as a collection of features (nodes) and links (edges) that connect pairs of nodes. The ‘guilt-by-association’ principle^73^ implies shared biology of pathways. Moreover, biological networks demonstrate scale-free behavior^74,75^ indicating that they have a relatively large number of low-connectivity nodes and only a few high-connectivity nodes, called ‘hubs,’ that are likely to be essential to network function. In the analysis of cellular proteins, IL-8 signaling, leucocyte extravasation signaling, ILK signaling, glucocorticoid receptor signaling and clathrin-mediated endocytic signaling demonstrated high-connectivity (Fig. 2). The overlapping pathway analysis for the BALF comparison of sarcoidosis and controls identified complex networks with a large number of nodes (Fig. 4). Several immune pathways such as IL-8, leukocyte extravasation signaling, B-cell receptor signaling, phagosome formation, and communication between adaptive and innate immune response signaling demonstrated high-connectivity with each other. Several signal transduction pathways were also highly-connected to immune pathways. Similarly, serval metabolic pathways were highly connected and NRF2 mediated antioxidant response was a ‘node’ that connected the metabolic pathways to immune pathways. Immune pathways were also connected to acute phase response signing mediated by complement and coagulation activation. In the overlapping canonical pathways analysis of BALF proteins in progressive and non-progressive cases, CD28, CDC-42 and IL-8 signaling, and Th1 pathways had high-connectivity suggesting a central role of these pathways in the progression of pulmonary sarcoidosis. Identifying networks of sarcoidosis development and progression in larger samples would allow data partition-based modeling approaches to reveal network topology and may provide valuable insights into disease biology that can not be revealed with conventional reductionist approaches.

Despite the small sample size, we believe our pilot study provides proof of concept for this line of investigation. Our experimental design is robust as we used stringent thresholds for protein identification and a conservative permutation test that decreased the chances of false positives to determine the differentially expressed cellular proteins. Similarly, for the BALF study, we examined each sample in triplicate. We also used a robust PECA procedure that implements algorithms that identifies peptide-level quantitative differences for more robust inferences regarding protein levels^76^. We expected that this mass spectrometry based bioinformatics workflow would provide a pipeline for application to future large-scale studies in sarcoidosis. A larger sample size would provide more robust inferences regarding the cellular mechanisms of progressive sarcoidosis in a cohort that represents heterogeneity in disease biology and yet allow implementation of resampling methods such as bootstrapping and cross validation for data analysis. We anticipated that workflows developed in this pilot study would identify pathways in peripheral blood mononuclear cells or lymph node tissue, some of which will overlap with the pathways in the lung, as well as some that might differ in direction between BAL and blood or be distinct and apparent in blood only, potentially serving as an easily accessible biomarker. Furthermore, we also hypothesized there would be activation of kinase signal transduction pathways after PBMC recruitment to the lung or other organ and activation of specific canonical and signaling pathways that would govern disease progression or remission.

## Conclusions and future directions

The pathophysiologic mechanisms that explain the variability in disease manifestations and course in sarcoidosis are not well understood. A significant challenge is the lack of established disease models that represent the systems contributing to the immune response in sarcoidosis. Single molecule studies are important for understanding the disease biology in sarcoidosis but fail to capture the interactions involved in heterogeneous diseases. Systems levels approaches will be critical to improve our understanding of sarcoidosis. As proteins are the primary effectors of cellular function, characterization of the changes in proteins will be essential to improve our knowledge of sarcoidosis. We established promising proteomics workflows that will be valuable to develop models (classifiers) for diagnosis and prognosis and also identify therapeutically tenable treatment targets in sarcoidosis. Investigating the cellular and BALF protein changes provides an opportunity to examine the complex interplay of protein interactions response for the development and progression of sarcoidosis as well test the validity of protein participating in these biological processes as biomarkers for disease diagnosis and predict progression. The novel mechanisms identified in our pilot study will need to be evaluated with conventional structure function study to determine causal links in sarcoidosis.

## Materials and methods

The study was approved by the University of Minnesota (UMN) IRB (protocol number 1501M60321and the National Jewish Health IRB (protocol number HS-2458) and all studies were conducted under the relevant guidelines/regulations. Study participants provided informed consent for the collection of BAL fluid and cells for these studies.

Eligible subjects consisted of individuals with sarcoidosis defined by the criteria outlined in the Joint Statement of American Thoracic Society (ATS), the European Respiratory Society (ERS) and the World Association of Sarcoidosis and Other Granulomatous Disorders (WASOG)^3^. Subjects without presence of another disease that could significantly affect patient immune response were also enrolled as healthy controls. Bronchoscopy and bronchoalveolar lavage were performed per standard protocol at UMN and NJH^77^. Four newly diagnosed sarcoidosis subjects were enrolled for examination of BAL cells at UMN (Table 1). Leftover BAL cells from four normal controls were obtained from prior research studies. For the BALF studies, 10 sarcoidosis subjects and 7 healthy controls were enrolled at NJH (Table 2). After collection, the BAL was transported on ice, centrifuged at 500*g* for 10 min, and the resulting cells and supernatant were stored at − 80 °C using common procedures at the two sites.

For the BALF studies, the sarcoidosis subjects were divided into two distinct phenotypes: those with non-progressive pulmonary disease and those with progressive pulmonary disease using criteria previously established^17,78,79^. Non-progressive pulmonary disease cases had stable disease and met the following criteria on up to two-years follow-up or more: (1) ≤ 10% decline in FVC or FEV1 and a ≤ 15% decline in DLCO and a stable CXR, and/or (2) ≥ 10% improvement in FVC or a ≥ 15% improvement in DLCO or improved CXR AND (3) no indication for immunosuppressive therapy. Progressive pulmonary disease cases met any of the following criteria from diagnosis/initial evaluation on at least two-year follow-up: (1) ≥ 10% decline in FVC and/or FEV1; or a ≥ 15% decline in DLCO; or (2) worsening CXR as determined by the interpreting radiologist/ investigator; and/or (3) start of immune-suppressive therapy.

### Protein isolation and MS spectral-data acquisition

#### Mixed BAL cells:

BAL cells were resuspended in hypotonic lysis buffer with HALT protease inhibitor cocktail (Thermo Fisher Scientific), and lysed using sequential cell disruption techniques including a freeze–thaw at 98 °C with vortexing and sonication (Sonics) on ice before buffering with 1 M triethylammonium bicarbonate (Sigma). The lysed cells were then centrifuged at 20,000*g* for 15 minutes and the supernatant was collected for further processing. To increase the protein recovery, the pellet from this step was resuspended in a buffer (containing 7 M urea and 2 M thiourea in 0.4 M triethylammonium bicarbonate at pH 8.5), freeze-thawed, vortexed and centrifuged at 15,000*g* for 15 min at 20 °C. The supernatants from the two centrifugation steps were combined and concentrated using an Amicon 3-MWCO filter (Millipore). An equal amount of protein was processed for in-gel cleanup and digestion (EMBL Method), reduced with dithiothreitol (Sigma-Aldrich), treated with iodoacetamide (Sigma-Aldrich) to block cysteine residues, digested with trypsin (Promega) and cleaned with an MCX stage tip (3M-Empore 2241). Isobaric labeling of digested peptides was carried out with TMT-10Plex (Thermo Fisher Scientific) reagent followed by MCX and SPE cleanup with appropriate buffer exchanges, and offline fractionation on Shimadzu Prominence with Xbridge 150 × 2.1 mm column (Waters) with two-minute fractions at a flow rate of 200µL/min, and peptide amounts of 15mAU-equivalent aliquots from fractions 7–38 were concatenated. LC–MS data was acquired for each concatenated fraction using an Easy-nLC 1,000 HPLC (Thermo Fisher Scientific, Waltham, MA) in tandem with an Orbitrap Fusion (Thermo Fisher Scientific) MS instrument.

### BALF proteins:

The BALF was processed using our previously published protocol^80,81^. Briefly, BALF was sonicated (Sonics), centrifuged for 15 min at 14,000*g* at 4 °C and filtered with pre-rinsed (5% methanol and water) syringe (Monoject, Covidien) and 0.22um PES filter to remove remaining particulates. The fluid was then concentrated and desalted using Amicon 3-MWCO filters, and a Bradford assay (Bio-Rad) was used to quantify protein. High-abundance proteins were removed using a Seppro IgY 14 spin-column (Sigma-Aldrich) with appropriate buffer exchanges. Equal amount of enriched medium- and low-abundance protein was processed for in-gel cleanup and digestion similar to the BAL cells above. LC–MS data was acquired for each concatenated fraction using an Easy-nLC 1,000 HPLC in tandem with an Orbitrap Fusion using settings similar to BAL cells analysis with minor differences. The differences were (1) The column was heated to 50 °C and (2) the dynamic exclusion was set to 15 s with a 10-ppm high and low mass tolerance.

### Mass spectral dataset analysis by sequence database search for protein identification and quantification

The BAL cell quantification was accomplished using TMT reagent, and the BALF dataset was analyzed using MS1 spectral quantification.

#### Identification and quantification of TMT-labeled cellular proteins:

The spectral dataset was searched against the target-decoy version of the Human UniProt database (72,886 protein sequences; October 10th 2018) along with the contaminant sequences from the cRAP database (https://www.thegpm.org/crap/). Scaffold Q + (version Scaffold_4.8.9, Proteome Software Inc., Portland, OR) was used to perform TMT-based peptide quantitation and protein identification. The threshold of peptide identifications was set at an FDR < 0.5% by the Scaffold Local FDR algorithm. The protein identifications were accepted if they could be established at greater than 99.0% probability and contained at least one peptide^82^. Channels were corrected according to the algorithm described in i-Tracker^83^. Normalization was performed iteratively (across samples and spectra) on intensities, as described in *Statistical Analysis of Relative Labeled Mass Spectrometry Data from Complex Samples Using ANOVA*^84^. Medians were used for averaging. Spectra data were log-transformed, pruned off those matched to multiple proteins and those missing a reference value, and weighted by an adaptive intensity-weighting algorithm. Of 23,837 spectra in the experiment at the given thresholds, 16,890 (71%) were included in quantitation. The proteins that matched to the cRAP or the decoy sequence were removed from analysis.

#### Identification and MS1quant label-free quantification of BALF proteins

Raw files were searched against the target-decoy version of Human UniProt database (73,928 protein sequences, November 21 2019) along with the cRAP database using the MaxQuant 1.6.10.43 algorithm. Default search parameters were used as follows: peptide spectral matching and proteins with 1% FDR modifications include fixed carbamidomethyl of C, variable oxidation of M, and N-terminal acetylation. BALF samples were quantified in label-free quantification (LFQ) mode, and spectra were “matched between runs”.

### Statistical analysis

The peptide-level data for the BALF was imported into the GalaxyP (https://galaxyp.org) framework for implementing the Peptide-level Expression Change Averaging (PECA)-procedure^76^ using the Bioconductor package (https://www.bioconductor.org/packages/release/bioc/html/PECA.html). This method differs from the common approach, where protein expression intensities are precomputed from the peptide data and an expression change between two groups of samples is first calculated for each measured peptide. The corresponding protein-level expression changes are then defined as medians over the peptide-level changes. For this analysis, we determined the modified t-statistic, which is calculated using the linear modeling approach in the Bioconductor limma (linear models for microarray data) package^85^. To identify differential expression in the BALF dataset, the comparability of relative expression changes between alternative peptides was investigated by considering signal log-ratios by a two-sample *t*-test with a p-value ≤ 0.05 corrected for multiple hypotheses testing. For the intracellular proteins, given the substantially higher number of proteins detected, we used a conservative permutation test to decrease the possibility of type 1 error rate with an unadjusted significance level p ≤ 0.05 corrected by the Benjamini–Hochberg method for testing multiple hypotheses.

To gain insight into the biological significance of differentially expressed proteins, we performed functional analysis using Ingenuity Pathway Analysis [IPA (IPA QIAGEN, Redwood City https://www.quiagen.com/ingenuty)]. This analysis was performed on proteins with an FDR corrected p-value ≤ 0.05 as the cutoff for differential expression for both BAL cell and fluid datasets. The IPA core analysis was performed using the difference of the weighted log fold change between comparison groups. We focused on canonical pathways that met a Benjamini and Hochberg (B-H)–corrected p-value obtained using the right-tailed Fisher exact test of ≤ 0.05 (equivalent to −log [B-H p-value] ≥ 1.3), as done previously^81^. We also examined on Overlapping Canonical Pathways functionality in IPA which is designed to visualize the shared biology in pathways through the common features (genes/proteins) in the pathways. The network of overlapping pathways shows each canonical pathway meeting the statistical threshold of −log (B-H p-value) ≥ 1.3 as a single “node”. An edge connects any two pathways when there are at least two common proteins shared between the pathways.

## Supplementary information

Supplementary information

Supplementary Table S1

Supplementary Table S2

Supplementary Table S3

## Data Availability

The MS proteomics datasets were deposited to the ProteomeXchange Consortium via the PRIDE partner repository with the dataset identifier PXD014438 (BAL cells) and PXD016637 (BALF).

## Abbreviations

BAL: Bronchoalveolar lavage
BALF: Bronchoalveolar lavage fluid
TNF: Tumor necrosis factor
TLR: Toll-like receptor
IL: Interleukin
SELDI: Surface-enhanced laser desorption ionization
TOF: Time-of-flight
MS: Mass spectrometer
MALDI: Matrix-assisted laser desorption ionization
2 DE: 2-Dimension electrophoresis
CXR: Chest X-ray
Treg: Regulatory T cells
FVC: Forced vital capacity
FEV: Forced expiratory volume
DLCO: Diffusing capacity for carbon monoxide
PI3K: Phosphoinositide 3-kinase
mTOR: Mammalian target of rapamycin complex 1
