# Supplementary material for: Novel protein pathways in development and progression of pulmonary sarcoidosis
Source: Sci Rep. 2020 Aug 6;10:13282. doi: 10.1038/s41598-020-69281-8 (PMC7413390; doi:10.1038/s41598-020-69281-8)
Supplement: Supplementary file 1 — Supplementary information [file 41598_2020_69281_MOESM1_ESM.docx]

**Novel Protein Pathways in Development and Progression of Pulmonary Sarcoidosis.**

Bhargava M*^1^, Viken KJ^1^, Barkes B^2^, Griffin TJ^3^, Gillespie M^2^, Jagtap PD^3^, Sajulga R^3^, Peterson EJ^4^, Dincer HE^1^, Li Li^2^, Restrepo CI^2^, O’Connor BP^5^, Fingerlin TE^5^, Perlman DM^1^, Maier LA^2^

^1^ Division of Pulmonary, Critical Care and Sleep Medicine, University of Minnesota, Minneapolis, MN; ^2^Division of Environmental and Occupational Health Sciences, National Jewish Health, Denver Colorado, ^3^ Biochemistry, Molecular Biology and Biophysics, College of Biological Sciences, University of Minnesota, Minneapolis MN, ^4^Center for Immunology, University of Minnesota, Minneapolis, MN, ^5^Center for Genes, Environment and Health, National Jewish Health, Denver, CO

This manuscript has supplemental data

Supplemental Table S1: BAL cellular protein dataset

Supplemental Table S2: BAL fluid protein dataset for identification

Supplemental Table S3: BAL fluid differentially expressed proteins
